# Therapeutic approaches in patients with bone metastasis due to endometrial carcinoma – A systematic review

**DOI:** 10.1016/j.jbo.2023.100485

**Published:** 2023-05-15

**Authors:** Martin Heidinger, Elisa Simonnet, Li Mei Koh, Brigitte Frey Tirri, Marcus Vetter

**Affiliations:** a Women’s Clinic, Cantonal Hospital Baselland, Rheinstrasse 26, 4410 Liestal, Switzerland; b University of Basel, Petersplatz 1, 4001 Basel, Switzerland; c Breast Center, University Hospital Basel, Spitalstrasse 21, 4031 Basel, Switzerland; d Medical Oncology, Cantonal Hospital Baselland, Medical University Clinic, Muehlemattstrasse 13, 4410 Liestal, Switzerland

**Keywords:** Bone metastasis, Endometrial neoplasms, Cytoreduction surgical procedures, Chemotherapy, Radiotherapy, Systematic review

## Abstract

**Background:**

Bone metastases (BM) are uncommon in endometrial carcinoma (EC), without information on the optimal oncologic management of patients with BM in EC. Here, we systematically review clinical characteristics, treatment approaches and prognosis in patients with BM in EC.

**Methods:**

We conducted a systematic literature search until 27th March 2022 on PubMed, MEDLINE, Embase and clinicaltrials.gov. Outcomes included treatment frequency and survival after BM with comparators being treatment approaches (local cytoreductive bone surgery, systemic therapy, and local radiotherapy). Risk of bias was assessed using the NIH Quality Assessment Tool and Navigation Guide methodology.

**Results:**

We retrieved 1096 records of which 112 retrospective studies (12 cohort studies, 12/12 fair quality; 100 case studies, 100/100 low quality) with a total of 1566 patients were included. The majority had a primary diagnosis of FIGO stage IV, grade 3 endometrioid EC. Singular BM were present in a median of 39.2%, multiple BM in 60.8% and synchronous additional distant metastases in 48.1% of patients respectively. In patients with secondary BM median time to bone recurrence was 14 months. Median survival after BM was 12 months. Local cytoreductive bone surgery was assessed in 7/13 cohorts and performed in a median of 15.8% (interquartile range [IQR] 10.3–43.0) of patients. Chemotherapy was assessed in 11/13 cohorts and administered in a median of 55.5% (IQR 41.0–63.9), hormonal therapy (7/13 cohorts) in 24.7% (IQR 16.3–36.0), and osteooncologic therapy (4/13 cohorts) in 2.7% (IQR 0.0–7.5) of patients respectively. Local radiotherapy was assessed in 9/13 cohorts and performed in a median of 66.7% (IQR 55.6–70.0) of patients. Survival benefits were seen in 2/3 cohorts after local cytoreductive bone surgery, and in 2/7 cohorts after chemotherapy without survival benefits in the remaining cohorts and investigated therapies. Limitations include the lack of controlled intervention studies, the heterogeneity and retrospective nature of the investigated populations.

**Conclusions:**

This systematic review shows heterogenous therapeutic approaches in clinical practice without clear evidence for optimal oncologic management for patients with BM in EC.

## Highlights

• Systematic review of 1566 patients with bone metastasis in endometrial carcinoma.
• Median survival after bone metastasis ranged from 10 to 16 months.
• Treatment approaches are heterogeneous.

## Graphical abstract

**Figure:**
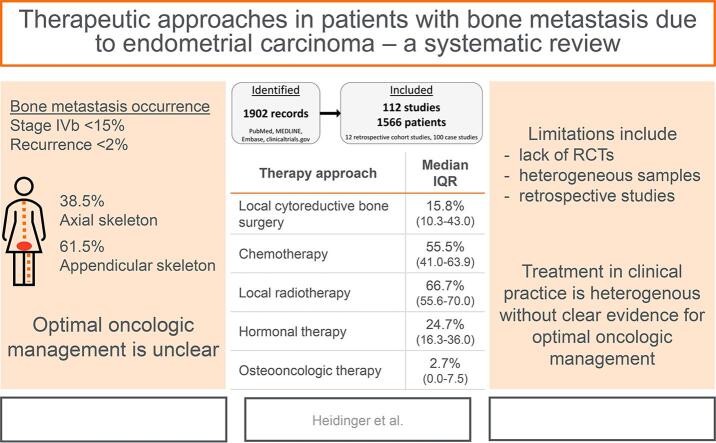

## 1 Introduction

Endometrial cancer (EC) is the sixth most common cancer in women worldwide and the most common gynecological cancer in Europe and North America, showing an increasing incidence over the past decade [1], [2], [3], [4], [5]. Prognosis for primary early-stage EC is generally considered good, however 3–13% of EC patients present with stage IV disease [6], [7], [8]. Furthermore, recurrence occurs in 13–18% of patients [9], [10], with approximately 8% presenting with distant recurrence [11]. Risk of recurrence increases with advancing stage at primary diagnosis from 6.5% in stage I up to 66.7% in stage IV [12]. Seventy percent of patients have a recurrence within the first 3 years after treatment [10], [13]. Prognostic estimations have increased in accuracy by the classification of ECs into four molecular subtypes. These were first published in 2013 based on non-metastatic EC samples within The Cancer Genome Atlas (TCGA) Research Network [14]. Whilst DNA polymerase epsilon (POLE) mutated (*POLE*mut) (also referred to as ultramutated) tumors are present in approximately 5–15% of patients and harbor the best clinical outcomes (disease specific survival at 48 months of 100%), EC with TP53 mutations (p53abn, also being named copy-number high serous-like) occur in approximately 10% of patients and show the worst overall and disease specific survival (DSS) (43% at 48 months), as well as the highest rates of locoregional and distant recurrences. Tumors of no-specific-molecular profile (NSMP) have also been described as copy-number low endometrioid-like and represent the largest cohort (up to 60%). Prognosis in NSMP EC has been described as intermediate, depending on stage at diagnosis, histologic grading, and additional prognostic factors. The fourth subtype is characterized through microsatellite instability (MSI, also described as mismatch-repair-deficient [dMMR]), which is present in one-quarter of cases. Clinical outcomes in MSI EC are also intermediate with slightly reduced survival outcomes compared to NSMP EC [14], [15], [16]. Despite new systemic treatment approaches including immunotherapy in advanced, metastatic and recurrent EC [17], [18], overall survival (OS) in recurrent as well as metastatic endometrial carcinoma is short, with median survival between 9 and 15 months [12], [19], [20] and 5-year OS in stage IVb EC reported between 15.3 and 20.1% [8], [21].

Even though bone is a common site of metastases in cancers such as breast, prostate, and lung [22], its involvement is generally considered rare in EC. Bone metastasis is generally considered to occur via hematogenous spread [23]. The reported clinical incidence rates in EC recurrence vary between 0.3 and 1.9% [11], [24], [25], [26], [27], [28], [29], [30], [31], whilst in stage IVb disease, up to 15% of patients are reported to have bone involvement [32]. Non-endometrioid histology has been shown to harbor an increased risk for BM compared to endometrioid EC [30]. Prolonged survival of EC patients as well as enhanced imaging techniques may increase the diagnosis of bone metastasis (BM), with post-mortem analysis reporting BM in up to 34.7% of EC patients [33]. Positron emission tomography-computed tomography appears to detect more BM compared to conventional computed tomography, with BM being predominantly reported as lytic lesions [34]. In general, patients with BM in EC have a higher risk for skeletal related events (SREs) and prognosis is significantly impaired after the occurrence of BM [35]. Currently, no information exists on the optimal oncologic management of patients with BM in EC.

Here, we conducted a systematic literature review on clinical characteristics, therapeutic approaches, and prognosis in patients with BM in EC.

## 2 Materials and methods

### 2.1 Eligibility criteria

This review aimed to find, assess, and synthesize all randomized controlled trials, observational studies (all types), cohort (longitudinal) studies, case-control studies, case reports or case series containing patients with BM in EC, with available information on patient and tumor characteristics, therapeutic interventions, and survival after BM. We analyzed the following interventions: local cytoreductive bone surgery, chemotherapy after BM, hormonal therapy after BM, osteooncologic therapy after BM or external beam radiotherapy to metastatic bone. Frequency of the employed treatment approaches was included as the primary outcome.

#### 2.1.1 Participants

Studies of patients with BM in EC, with available information on therapeutic interventions and survival were eligible for this review. Patients with additional metastatic sites were also included.

#### 2.1.2 Interventions

We included: local cytoreductive bone surgery, chemotherapy after BM, hormonal therapy after BM, osteooncologic therapy after BM or external beam radiotherapy to metastatic bone.

#### 2.1.3 Comparators

Studies with the following comparator groups were eligible: no local cytoreductive bone surgery, no chemotherapy after BM, no hormonal therapy after BM, no osteooncologic therapy after BM or no external beam radiotherapy to metastatic bone.

#### 2.1.4 Outcomes

The investigated primary outcomes were frequency of the employed treatment approaches in patients with BM in EC. Secondary outcomes included frequency of treatment approaches based on patient and tumor characteristics and survival after BM.

#### 2.1.5 Study design

The following study designs were included: randomized controlled trials, observational studies (all types), cohort (longitudinal) studies, case-control studies, case reports or case series.

### 2.2 Search strategy

The detailed search string is presented in Appendix A. The search string was constructed by a medical doctor and health librarian. The following automation tools were used in the design of the search: Polyglot Search Translator [36], SearchRefinery [37] and The Systematic Review Accelerator [38]. We conducted searches in the following databases: PubMed, MEDLINE via Ovid, Embase via Elsevier and clinicaltrials.gov. Searches were run for all registered literature until 27th March 2022. All publication types were included in the search. We restricted our search to include the following languages: English, German, French, Spanish and Italian. We also manually checked the reference lists of the included studies and used the similar articles feature of the databases.

### 2.3 Study screening and selection

#### 2.3.1 Screening

**Fig. 1:**
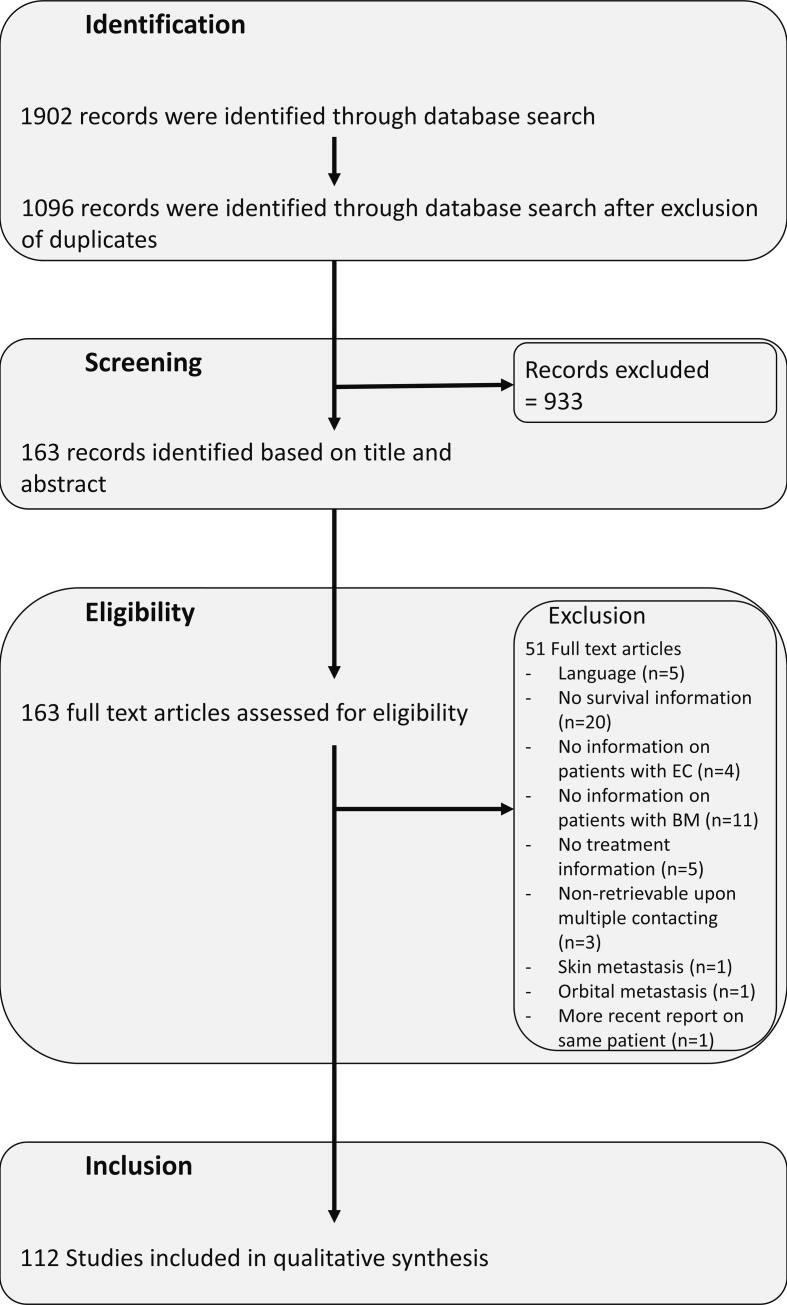
Preferred Reporting Items for Systematic Reviews and Meta-Analyses (PRISMA) flowchart for the selection of studies.

Two review authors (MH, ES) screened the titles and abstracts against the inclusion criteria. Once the initial title/abstract screening was completed, the full texts of the included studies from that stage were reviewed by two authors (MH, ES) to determine if they should be included. Discrepancies were resolved by referring to a third author or by consensus. Case reports were explicitly checked for follow-up reports of the same patient, and if available only the latest report was included. Available preprints were screened and reviewed, and studies included if publication of the final manuscript occurred before August 1st, 2022. Screenatron/Disputatron automation tool was used to screen articles [38]. Fig. 1 shows the PRISMA flow diagram for the selection process. Excluded articles and reasons for exclusion are listed in Appendix B.

#### 2.3.2 Data extraction

We used a data extraction form for study characteristics and outcome data, which was piloted on 50 studies in the review. One study author (MH) extracted the following data from included studies:

• types: randomized controlled trials, observational studies (all types), cohort (longitudinal) studies, cohort studies, case-control studies, case reports, case series;
• methods: year, study design, duration of follow-up;
• participants: age, histologic tumor subtype, grade, FIGO stage, BM status, distant metastasis (DM) status;
• interventions and comparators: (no) local cytoreductive bone surgery, (no) chemotherapy after BM, (no) hormonal therapy after BM, (no) osteooncologic therapy after BM, or (no) external beam radiotherapy to metastatic bone;
• outcomes: frequency of the employed treatment approaches in patients with BM in EC; frequency of treatment approaches based on patient and tumor characteristics; survival after bone metastasis.

### 2.4 Risk of bias Assessment

Two review authors (MH, ES) assessed the risk of bias for each study using the NIH Quality Assessment Tool for Observational Cohort Studies [39] and an adapted Navigation Guide methodology according to Nambiema et al. [40].

### 2.5 Data analysis

Patient characteristics of the study population and effect estimates are summarized using descriptive statistics with median and interquartile range (IQR) being calculated from the point values of investigated characteristics of the individual studies. For all studies reporting medical histories, therapeutic interventions, and survival of individual patients within retrospective cohort studies, univariate hazard ratios (HR) for survival after bone metastasis diagnosis were computed if not reported in the original published manuscript. The event of interest was time to death after occurrence of BM with right censoring at time of last reported follow-up. Univariate HR with corresponding 95% confidence intervals (CI) were estimated using the Kaplan-Meier method, log-rank test, and Cox regression analysis. All statistical analyses were conducted using R (version 4.03) in the RStudio environment.

## 3 Results

### 3.1 Study selection

We initially retrieved 1902 records through database search, representing 1096 records after exclusion of duplicates. No registered or ongoing clinical trials were found. After screening titles and abstracts, 163 records were retrieved for full-text screening. Finally, we included 112 studies (Appendix C) in this review, with a detailed flow-chart depicted in Fig. 1.

### 3.2 Risk of bias in studies

Of the included studies, 12 studies were retrospective cohort studies and 100 were case studies. We used the NIH Quality Assessment Tool for Observational Cohort Studies [39] to assess the risk of bias in all retrospective cohort studies. We rated all included studies as fair quality, with the principal missing quality assessment criteria being sample size justification (12/12), absence of blinded outcome assessors due the exposure status (12/12), and the lack of clearly defined and consistent exposure measures (7/12) (Table S1). Case series and Case reports were assessed according to the adapted Navigation Guide methodology [40]. All case series and case reports were rated as low quality. The principal quality shortcomings were lack of accuracy in exposure/intervention assessment (53/100), and inadequate outcome measures (26/100) as depicted in Table S2.

### 3.3 Study characteristics

Characteristics of the included studies, regarding year of publication, location and time period, study type, included patient and cohort characteristics, time to BM, OS and survival after BM are described in Table 1. The 12 included retrospective cohort studies were published between 2010 and 2022 and covered a treatment time from 1984 to 2019. Characteristics of cases published as case reports and case series are documented in Table S3, with a compound presentation included in Table 1. The included case studies were published between 1950 and 2022 and covered a treatment time from 1933 to 2021.

**Table 1:** Study, patient, and survival characteristics of cohorts with bone metastasis in endometrial carcinoma in the included studies.

**Reference**	**Study location and period**	**Type of study**	**Number of patients**	**Cohort characteristics**	**Time to BM in patients with bone recurrence**	**Overall survival**	**Survival after bone metastasis**
Kehoe et al. 2010 [24]	Memorial Sloan Kettering Cancer Center, New York, USA 1990–2008	Retrospective cohort study	6144 patients w EC 21 patients w BM in EC - 15 patients w bone recurrence - 6 patients with BM at EC diagnosis	Age, median [range], years: 60 [32–84] FIGO stage at diagnosis: I n = 3; II n = 1; III n = 6; IV n = 8; n.A. n = 3 Histopathology: Adenocarcinoma n = 19; Clear cell carcinoma n = 1 Grade: G1 n = 3; G2 n = 5; G3 n = 12 Molecular subtype: n.A. Singular BM n = 3; Multiple BM n = 18 Other DM n = 13 (including lung, pelvis, lymph nodes, vagina)	Months, median [range]: 10 [3–148]	Entire cohort w BM - months, median: 25 Cohort w bone recurrence in EC - months, median (95 %CI): 32 (14–49) Cohort w BM at EC diagnosis - months, median (95 %CI): 17 (2–32)	Entire cohort w BM – months, median [range]: 10 [1–199] Cohort w bone recurrence in EC – months, median (95 %CI): 10 (5–15) Cohort w BM at EC diagnosis – months, median (95 %CI): 17 (2–32)
Blecharz et al. 2011 [26]	Maria Skłodowska-Curie, Memorial Institute, Kraków, Poland January 1985-June 2005	Retrospective cohort study	1610 patients with stage I-II EC 110 patients w hematogenous metastasis - 23 patients w bone recurrence in EC	Age, median [range], years in total cohort (n = 110) – 62.5 [40–83] Age in BM cohort: n.A. FIGO stage at diagnosis n.A. Histopathology: Adenocarcinoma n = 23 Grade: n.A. Molecular subtype: n.A. Singular BM n = 6; Multiple BM n = 17 Other DM n = 13 (including lung, liver)	Months, median [range]: 26 [5–36]	n.R.	12-month survival rate: 21.7% 26-month survival rate: 8.7% 60-month survival rate: 8.7%
Uccella et al. 2013 [25]	Mayo Clinic, Rochester, Minnesota, USA 1984–2001	Retrospective cohort study	1632 patients w EC 19 patients w BM in EC - 16 patients w bone recurrence - 3 patients with BM at EC diagnosis	Age, median [range], years: 65 [47–80] FIGO stage at diagnosis: I n = 10; II n = 1; III n = 2; IV n = 5; n.A. n = 1 Histopathology: Adenocarcinoma n = 14; Adenosquamous carcinoma n = 2; Serous carcinoma n = 3 Grade: G1 n = 1; G2 n = 6; G3 n = 12 Molecular subtype: n.A. Singular BM n = 13; Multiple BM n = 6 Other DM n = 9 (including lung, liver, lymph nodes, brain, intraperitoneal spread)	Months, median [range]: 19.5 [3–114]	Months, median [range]: 48 [2–267]	Months, median [range]: 12 [2–267]
Yoon et al. 2014 [41]	Samsung Medical Center, Seoul, South Korea October 1994-May 2012	Retrospective cohort study	1185 patients w EC 22 patients w BM in EC (1 excluded from survival analysis due to concomitant breast cancer) - 17 patients w bone recurrence - 4 patients with BM at EC diagnosis	Age, median [range], years: 59 [28–72] FIGO stage at diagnosis: I n = 3; II n = 1; III n = 10; IV n = 7 Histopathology: Endometrioid n = 8; Adenocarcinoma not further specified n = 4; Serous carcinoma n = 5; Clear cell carcinoma n = 1; villoglandular carcinoma n = 1; poorly differentiated carcinoma n = 2 Grade: G1 n = 1; G2 n = 4; G3 n = 10; n.A. n = 6 Molecular subtype: n.A. Singular BM n = 10; Multiple BM n = 11 Other DM n = 13 (including lung, liver, lymph nodes, brain)	Months, median [range]: 9 [2–43]	Entire cohort w BM – months, median [range]: 33 [9–57] Cohort w bone recurrence in EC – months, median: 36 Cohort w BM at EC diagnosis – months, median: 13	Months, median [range]: 15 [12–17]
Kimyon et al. 2016 [28]	Etlik Zubeyde Hanim Women’s Health Teaching and Research Hospital, Ankara, Turkey January 1993-May 2013	Retrospective cohort analysis	1345 patients w EC, HE and BSO, and available follow-up data 10 patients w bone recurrence in EC - 1 patient without available information on recurrence treatment excluded	Age, median [range], years: 62 [51–71] FIGO stage at diagnosis: I n = 5; II n = 0; III n = 4; IV n = 0 Histopathology: Adenocarcinoma n = 6; Serous carcinoma n = 1; Clear cell carcinoma n = 1; Undifferentiated carcinoma n = 1 Grade: G1 n = 2; G2 n = 4; G3 n = 3 Molecular subtype: n.A. Singular BM n = 5; Multiple BM n = 4 Other DM n = 2 (including lung, brain, intraperitoneal spread)	Months, median [range]: 14 [1–68]	Months, median [range]: 24 [6–115]	Months, median [range]: 7 [2–47]
Liu et al. 2019 [35]	SEER database USA 2010–2014	Retrospective cohort analysis	2948 patients w stage IV EC including endometrioid, non-endometrioid, and sarcoma histology 1152 patients w distant metastases at diagnosis of EC - 270 patients w BM in EC - 110 patients w BM and no other DM	Age < 65 years in patients w BM and no other DM: n = 48 Histopathology in patients w BM: Endometrioid n = 92; Non-Endometrioid n = 111; Sarcoma n = 63; n.A. n = 4 Grade in patients w BM: G1 and G2 n = 44; G3 and G4 n = 222; n.A. n = 4 Molecular subtype: n.A. Singular vs. multiple BM: n.A. Other DM n = 169 (including lung, liver, brain)	n.A.	Months, median: 6	n.A.
Ouldamer et al. 2019 [11]	FRANCOGYN database France 2001–2013	Retrospective cohort analysis	1444 patients w presumed early-stage EC - 110 patients w any distant recurrence - 10 patients w bone recurrence	Age, mean [range], years: 75.5 [66–87] FIGO stage at diagnosis: n.A. Histopathology: n.A. Grade: G1 n = 1; G2 n = 3; G3 n = 6 Molecular subtype: n.A. Singular vs. multiple BM: n.A. Other DM n.A.	Months, median: 19	n.R.	3-year survival rate: 37.3%
McEachron et al. 2020 [29]	SUNY Downstate Medical Center Good Samaritan Hospital Medical Center New York Presbyterian – Brooklyn Methodist Hospital New York, USA 2012–2019	Retrospective cohort analysis	1085 patients w EC 10 patients w BM in EC - 8 patients w bone recurrence - 2 patients with BM at EC diagnosis	Age, median [range], years: 65 [31–71] FIGO stage at diagnosis: I n = 2; II n = 0; III n = 5; IV n = 3 Histopathology: Endometrioid n = 7; Serous carcinoma n = 3 Grade: G1 n = 0; G2 n = 2; G3 n = 5; n.A. n = 3 Molecular subtype: MSI n = 7 Singular BM n = 3; Multiple BM n = 7 Other DM n = 8 (including lung, liver, intraperitoneal spread, vagina, parametria; excluding pelvic and paraaortic lymph nodes)	Months, median [range]: 12 [4–44]	Months, median [range]: 21.5 [6–66]	Months, median [range]: 10 [3–22]
Guo et al. 2020 [32]	SEER database USA 2010–2015	Retrospective cohort analysis	730 patients w stage IVb EC at diagnosis w at least 1 DM site including lung, bone, or brain; including endometrioid, serous, or clear cell histology - 113 patients w BM at EC diagnosis	Age in total cohort (n = 730), median: 64.81 ± 10.92 Histopathology in total cohort (n = 730): Endometrioid n = 575; Non-Endometrioid n = 155 Grade in total cohort (n = 730): G1 n = 55; G2 n = 109; G3 n = 360; n.A. n = 206 Molecular subtype: n.A. Singular vs. multiple BM: n.A. Other DM in total cohort (n = 730): n = 218	n.A.	Months, median (95 %CI): 15 (8.55–21.45)	n.A.
Hu et al. 2021 [42]	SEER database USA 2010–2016	Retrospective cohort analysis	584 patients w EC and BM at diagnosis including endometrioid, non-endometrioid, and sarcoma histology	Age, median, years: 64 Histopathology: Endometrioid n = 211; Non-Endometrioid n = 301; Sarcoma n = 72 Grade: G1 and G2 n = 76; G3 and G4 n = 329; n.A. n = 179 Molecular subtype: n.A. Singular vs. multiple BM n.A. Other DM n ≥ 270	n.A.	1-year overall survival rate: 33.8%	n.A.
Yang et al. 2021 [30]	SEER database USA 2010–2015	Retrospective cohort analysis	54 077 patients with EC, complete clinicopathologic features, demographic data, and follow-up information - 364 patients w BM at EC diagnosis	Age < 67 years in patients w BM at EC (n): 220 Histopathology in patients w BM at EC: Endometrioid n = 116; Non-Endometrioid n = 163; Sarcoma n = 85 Grade in patients w BM at EC: G1 n = 16; G2 n = 43; G3 n = 193; G4 n = 112 Molecular subtype: n.A. Singular vs. multiple BM: n.A. Other DM n ≥ 175	n.R.	n.R.	n.R.
Wang et al. 2022 [31]	Peking University People’s Hospital, Beijing (Beijing/Peking), China 2000–2019	Retrospective Cohort Analysis	1662 patients w EC 14 patients w BM in EC (1 excluded from survival analysis due to missing follow-up information) - 9 patients w bone recurrence - 4 patients with BM at EC diagnosis	Age, median [range], years: 58 [45–76] FIGO stage at diagnosis: I n = 2; II n = 1; III n = 1; IV n = 5; n.A. n = 4 Histopathology: Endometrioid n = 12; Clear cell carcinoma n = 1 Grade: G1 n = 1; G2 n = 3; G3 n = 4; n.A. n = 5 Molecular subtype: n.A. Singular BM n = 4; Multiple BM n = 9 Other DM n = 6 (including lung, intraperitoneal spread, skin)	Months, median [range]: 13 [5–144]	Months, median [range]: 40 [10–160]	Months, median [range]: 16 [1–57]
Case reports as outlined in table S3	1933–2021	Case Reports	107 patients w BM in EC - 63 patients w bone recurrence - 44 patients with BM at EC diagnosis	Age, median [range], years: 62 [25–87] FIGO stage at diagnosis: I n = 29; II n = 6; III n = 15; IV n = 44; n.A. n = 13 Histopathology: Adenocarcinoma n = 75; Non-Adenocarcinoma n = 24; n.A. n = 8 Grade: G1 n = 15; G2 n = 28; G3 n = 41; n.A. n = 23 Molecular subtype: NSMP n = 1 Singular BM n = 62; Multiple BM n = 43; n.A. n = 2 Other DM Yes n = 45; No n = 62	Months, median [range]: 17 [1–220]	Months, median [range]: 25 [1–264]	Months, median [range]: 12 [1–145] 12-month survival-rate (95 %CI): 51.4% (42.0–60.7) 60-month survival-rate (95 %CI): 6.5% (3.2–12.9)

BM – bone metastasis; EC – endometrial carcinoma; w – with; HE – hysterectomy; BSO – bilateral salpingo-oophorectomy; DM – distant metastasis; CI – confidence interval; n.R. – not reported; n.A. – not available; MSI – microsatellite instability; NSMP – no-specific-molecular-profile.

A total of 1566 patients with BM in EC from the 112 studies were analyzed. The median age ranged from 58 to 65 years (Table 1). Median FIGO stage at diagnosis of endometrial carcinoma in patients sustaining a bone metastasis over the included studies was 20% for stage I (IQR 14.8–36.9), 4.8% (IQR 2.4–5.6) for stage II, 28.6% (IQR 12.7–43.8) for stage III, and 33.3% (IQR 28.2–38.3) for stage IV, excluding the three retrospective cohort studies, in which only patients with stage IV at diagnosis were included. Grading of the endometrial carcinoma was 7.7% on median for grade I (IQR 5.0–14.6), 23.8% (IQR 21.5–29.7) for grade II, and 47.6% (IQR 32.7–53.6) for grade 3. One study reported to have 30.8% grade 4 carcinomas [30]. Two studies reported combined ratios of grade 1 and 2 endometrial carcinomas, with 13.0% [42] and 16.3% [35], as well as combined ratios for grade 3 and 4 endometrial carcinoma with 56.3% [42] and 82.2% [35] respectively. Molecular tumor information was available for 9 patients, with 8 exhibiting MSI and one being NSMP. Singular bone metastases were reported in a median of 39.2% (IQR 29.0–52.1) over the included studies, whilst multiple BM were present in a median of 60.8% (IQR 39.9–71.0). Information on localization of bone metastases was available for 202 patients with 273 metastatic sites [25,29,31,41, Studies of Table S3]; 61.5% (168/273) being metastases in the appendicular skeleton (33.3% [91/273] lower extremities, 23.4% [64/273] pelvic bones, 4.8% [13/273] upper extremities), and 38.5% (105/273) in the axial skeleton (25.6% [70/273] vertebral, 8.1% [22/273] ribs and sternal bone, 4.8% [13/273] skull). At least one other site of DM was present in a median of 48.1% (IQR 40.5–59.2).

Information on primary treatment for EC was available for 134 patients with bone recurrence [24,26,29,41, Studies of Table S3]. Of those, 93.3% (125/134) had received gynecologic surgery for EC (i.e., hysterectomy, bilateral salpingo-oophorectomy, pelvic and/or *para*-aortic lymphadenectomy), 26.1% (35/134) adjuvant chemotherapy, 62.7% (84/134) whole pelvic radiotherapy, and 38.1% (51/134) vaginal brachytherapy. Detailed results on primary therapy for EC in patients with recurrent BM is depicted in Table S4.

In patients with bone recurrence in EC, the median time to recurrence was 14 months (IQR 12–19) and ranged from 9 to 26 months in the included studies. Median overall survival after EC diagnosis in the investigated cohorts was 25 months (IQR 21.5–33, range 6–48). The median survival after BM in EC was 12 months and ranged from 10 to 16 months. Median survival after BM was 14 months (IQR 9–17, range 6–98) in patients with BM at EC diagnosis, and 11 months (IQR 11–13, range 7–16) in patients with BM recurrence.

Therapeutic approaches for bone metastasis include surgery, systemic therapy as well as radiotherapy, and are summarized in Table 2. Local cytoreductive surgery of bone metastasis was performed in a median of 15.8% of cases (IQR 10.3–43.0) over the investigated studies. Systemic therapies comprised of chemotherapy, which was performed on median in 55.5% of patients (IQR 41.0–63.9). Of the 49 patients with available detailed information on the used chemotherapeutic regimens, 79.6% (39/49) received platin-based chemotherapy and 6.1% (3/49) immunotherapy, as depicted in Table 3 [28,29, Studies of Table S3]. Hormonal therapy (median 24.7%, IQR 16.3–36.0) comprising progesterone derivatives and osteooncologic therapy (median 2.7%, IQR 0.0–7.5) comprising bisphosphonates and RANK-Ligands were performed less frequently. No information on systemic treatment sequence was available. Local radiotherapy to metastatic bone was administered in a median of 66.7% (IQR 55.6–70.0).

**Table 2:** Treatment frequency in patients with bone metastasis in endometrial carcinoma.

**Study**	**Local cytoreductive bone surgery**	**Chemotherapy after BM**	**Local radiotherapy for BM**	**Hormonal therapy after BM**	**Osteooncologic therapy after BM**
Kehoe et al. 2010	42.9% (9/21)	57.1% (12/21)	66.7% (14/21)	19.1% (4/21)	n.A.
Blecharz et al. 2011	0% (0/23)	73.9–100%*	73.9% (17/23)	30.4–65.2%*	n.A.
Uccella et al. 2013	15.8% (3/19)	10.5% (2/19)	73.7% (14/19)	52.6% (10/19)	5.3% (1/19)
Yoon et al. 2014	9.5% (2/21)	61.9% (13/21)	52.4% (11/21)	n.A.	n.A.
Kimyon et al. 2016	11.1% (1/9)	44.4% (4/9)	55.6% (5/9)	22.2% (2/9)	n.A.
Liu et al. 2019	n.A.	n.A.	n.A.	n.A.	n.A.
Ouldamer et al. 2019	n.A.	0% (0/10)	70% (7/10)	n.A.	n.A.
McEachron et al. 2020	n.A.	70% (7/10)	70% (7/10)	0% (0/10)	0% (0/10)
Guo et al. 2020	n.A.	n.A.	n.A.	n.A.	n.A.
Hu et al. 2021	n.A.	53.8% (314/584)	n.A.	n.A.	n.A.
Yang et al. 2021	n.A.	58.5% (213/364)	n.A.	n.A.	n.A.
Wang et al. 2022	100% (13/13)	30.8% (4/13)	23.1% (3/13)	7.7% (1/13)	0% (0/13)
Case Reports 1950–2022	43.0% (46/107)	50.5% (54/107)	60.8% (65/107)	27.1% (29/107)	14.0% (15/107)

BM – bone metastasis; n.A. – not available.

*not clearly stated in manuscript.

**Table 3:** Chemotherapeutic regimens of 49 patients with bone metastases in endometrial carcinoma.

Treatment	n	%
Platinum + Taxol based	28	57.1%
Platinum + Epirubicin or Doxorubicin	6	12.3%
Platinum + other agent	5	10.2%
Doxorubicin	5	10.2%
Immunotherapy	3	6.1%
Other	2	4.1%

Detailed information on treatment frequencies in patients with singular BM, multiple BM, as well as in patients with extraosseous disease could be extracted from six study samples, comprising 173 patients [21,22,25,26,28, Studies of Table S3] and are depicted in Table 4.

**Table 4:** Treatment frequencies in patients with singular bone metastasis, multiple bone metastases, and additional extraosseous distant metastasis in endometrial carcinoma.

	**Singular BM**	**Multiple BM**	**Additional extraosseous distant metastasis**
	n = 55	n = 39	n = 79
	Median, [IQR]	Median, [IQR]	Median, [IQR]
Local cytoreductive bone surgery	50.0% [22.2–54.1]	27.2% [6.3–44.9]	23.6% [2.8–37.9]
Chemotherapy after BM	45.9% [33.3–75.0]	50.0% [12.5–56.6]	50.0% [25.0–52.4]
Local radiotherapy for BM	62.2% [50.0–66.7]	72.4% [53.1–95.6]	51.9% [47.6–55.1]
Hormonal therapy after BM	30.2% [26.5–41.7]	0.0% [0.0–0.0]	27.7% [0.0–44.4]
Osteooncologic therapy after BM	0.0% [0.0–4.1]	0.0% [0.0–0.0]	0.0% [0.0–11.1]

BM – bone metastasis.

Treatment-specific survival information after BM were available for 8 patient cohorts and are summarized in Table 5. Survival estimates after local cytoreductive bone surgery are depicted for three patient samples. Two cohorts showed survival estimates in favor of this therapeutic approach (HR 0.31 and 0.32), whilst one showed an indifferent result (HR 1.15). Chemotherapeutic regimens were evaluated in seven samples. Two cohorts showed results favoring chemotherapy (HR 0.38 and 0.45), three samples showed trends towards a beneficial outcome (HR 0.51, 0.62, and 0.77), and in two samples indifferent results were shown (HR 0.98 and 1.02). Local radiotherapy to the metastatic bone was evaluated in 6 patient samples, with one showing trends towards survival benefits (HR 0.68), and 5 showing equivocal survival estimates (HR 0.47, 0.73, 0.94, 0.94, and 3.14). In three studies the use of hormonal therapy after bone metastasis could be evaluated, with two studies showing a trend towards a survival benefit (HR 0.51 and 0.51), and one study showing a survival disadvantage (HR 12.22). Finally, osteooncologic therapy was evaluated in two patient samples, both showing indifferent results (HR 0.85 and 3.87).

**Table 5:** Treatment-specific univariate hazard ratios for survival after bone metastasis in patients with endometrial carcinoma.

**Study**	**Local cytoreductive bone surgery HR (95 %CI)**		**Chemotherapy after BM HR (95 %CI)**		**Local radiotherapy for BM HR (95 %CI)**		**Hormonal therapy after BM HR (95 %CI)**		**Osteooncologic therapy after BM HR (95 %CI)**	
	Yes	No	Yes	No	Yes	No	Yes	No	Yes	No
Kehoe et al. 2010	0.31 (0.11–0.90)	Ref = 1	1.01 (0.39–2.61)	Ref = 1	0.73 (0.27–1.99)	Ref = 1	n.A.*		n.A.	
Uccella et al. 2013	1.15 (0.32–4.06)	Ref = 1	0.51 (0.07–3.94)	Ref = 1	0.94 (0.32–2.75)	Ref = 1	0.51 (0.19–1.40)	Ref = 1	3.87 (0.45–33.49)	Ref = 1
Kimyon et al. 2016	n.A.		0.77 (0.11–5.52)	Ref = 1	3.14 (0.32–30.56)	Ref = 1	12.22 (1.03–144.9)	Ref = 1	n.A.	
McEachron et al. 2020	n.A.		0.62 (0.10–3.87)	Ref = 1	0.47 (0.04–5.31)	Ref = 1	n.A.		n.A.	
Hu et al. 2021	n.A.		0.38 (0.31–0.45)	Ref = 1	n.A.		n.A.		n.A.	
Yang et al. 2021	n.A.		0.44 (0.33–0.58)	Ref = 1	n.A.		n.A.		n.A.	
Wang et al. 2022	n.A.		n.A.		0.94 (0.10–8.48)	Ref = 1	n.A.		n.A.	
Case Reports 1950–2022	0.32 (0.16–0.67)	Ref = 1	0.98 (0.53–1.85)	Ref = 1	0.68 (0.36–1.29)	Ref = 1	0.51 (0.24–1.08)	Ref = 1	0.85 (0.30–2.40)	Ref = 1

HR – hazard ratio; CI – confidence interval; Ref – reference; BM – bone metastasis; n.A. – not available.

*History of hormonal therapy was not reported for singular patients.

## 4 Discussion

Bone metastasis in EC is rare compared to other tumor types as e.g. breast or lung cancer, showing however the same multitude of therapeutic options. In this systematic review, we include a detailed characterization of patient and tumor characteristics for BM in EC, we present applied treatment concepts according to metastatic tumor burden and descriptively correspond the latter to survival after BM.

In the investigated studies, secondary occurrence of BM was shown to develop mainly within the first two years, corresponding to published results of temporal recurrence trends [10], [11]. In general, BM most commonly affect the axial skeleton and may cause SREs [22]. One previous analysis of BM in EC has shown 58.6% (17/29) of BM occurring in the axial skeleton [25]. Interestingly, in our review localization of BM were mainly reported in the appendicular skeleton (61.5%, 168/273). The generalizability of these data may be limited due to the limited representativeness of the sample, however it constates the biggest reported sample size to the best of our knowledge. Furthermore, 61% of patients showed multiple BM and approximately half of all patients had at least one other distant organ metastasis. Both multiple BM and extraosseous spread have been associated with shortened survival [11], [30], [32], [43]. With 26.7% of recurrences, DM were shown to be the most frequent first site of recurrence in the PORTEC-3 cohort, with a total median survival after the occurrence of DM of 1.4 years [44]. In an analysis of the SEER database Li et al. showed, that patients with stage IVb EC with organ metastases had significantly worse overall as well as cause-specific survival compared to peritoneal spread. Bone and brain metastases conferred to the worst prognosis, with OS in single-site metastasis of 18 and 6 months respectively [43]. In the analyzed cohorts of this systematic review, median survival ranged from 10 to 16 months after BM occurrence. However, even in metastatic disease cancer survivors with survival up to 267 months were reported [25].

Treatment of patients with BM in EC showed great heterogeneity. Currently no clear treatment recommendations are available for patients with BM in EC. Indications for the treatment of metastatic lesions include (i) the intent to cure, (ii) the intent to prolong survival, or (iii) to achieve local disease and symptom control for palliative care [22], [45]. The disease extent should be evaluated regarding (i) abdominal vs. distant metastases, (ii) isolated vs. disseminated metastases, (iii) de novo progression/metastasis vs. persistent progression/metastasis [46]. In general, patients with stage IVb disease or residual disease in stage III or IVa represent a unique prognostic risk group irrespective of molecular subtype [47]. Treatment options for patients with stage IVb EC regardless of molecular features include surgery if optimal cytoreduction being defined as macroscopic complete resection is feasible with acceptable morbidity and/or radiotherapy with or without chemotherapy. Neoadjuvant treatment using platinum-based chemotherapy or progestin derivates are suggested for unresectable recurrent or advanced disease [5], [46], [47]. Depending on response, subsequent surgery or definitive radiotherapy are recommended [47]. Recently, the concept of oligometastatic disease has emerged, which generally refers to patients with 1–5 metastases or sites of recurrence for which treatment approaches could be curative [45], [48], [49], [50].

In this systematic review, we analyzed local ablative treatment approaches, namely local cytoreductive bone surgery and local radiotherapy, as well as systemic therapies and found that survival rates were variable after the occurrence of BM.

### 4.1 Local ablative therapy

Local ablative therapies in metastatic disease have mainly been described as symptomatic treatment. The most common SREs in patients with BM are pain and structural bone damage possibly causing pathologic fractures, spinal cord compression, as well as reduced functionality and mobility. These SRE may require local therapies such as surgical intervention and/or local radiotherapy [22], [51]. In the present systematic review, we did not include SREs as outcomes. A previous systematic review investigated SREs in patients with BM with gynecologic malignancy comprising EC, however no comprehensive evidence on the type and frequency of SREs could be gathered [52]. This lack of information on the natural history of the disease represents a significant deficiency in this particular area.

In other cancers bone metastasectomy has been shown to have favorable outcomes for local control and survival. In differentiated thyroid cancer bone metastasectomy has been shown to be a significant factor for prolonged survival with complete resections showing the lowest local recurrence and highest survival rates [53], [54], [55], [56], [57], [58]. In addition, BM metastasectomy in lung cancer patients has been described as potentially prolonging survival [59], [60], [61]. Furthermore, local metastasectomy has been discussed for both pulmonary and liver metastasis in EC [62], [63], [64], [65], [66], [67], [68]. Liver metastasectomy has been shown to improve survival outcomes in combination with chemotherapy [66]. Three small series including patients with EC and lung metastasis have described improved survival rates after pulmonary metastasectomy [62], [69], [70]. One study by Dowdy et al. including 82 EC patients with pulmonary recurrences did not show a survival benefit by thoracotomy compared to hormonal treatment, whilst chemotherapy was associated with worse outcomes in this study [65]. Finally, local cytoreductive bone surgery has been suggested to enhance survival in EC patients with BM [71].

In the present samples the performance of local cytoreductive bone surgery in patients with singular BM seemed numerically higher (50.0%), compared to patients with multiple BM (27.2%) or additional extraosseous DM (23.6%). In two cohorts, comprising a total of 128 patients, a survival benefit was seen, whilst one cohort (n = 19) showed an equivocal result. Multiple BM were present in 41% and 86% within the two cohorts showing a survival benefit from local cytoreductive bone surgery, and in 32% of the cohort with equivocal results. Additional extraosseous DM were present in 42%, 62%, and 48% respectively. In a case series presented by Wang et al. local cytoreductive bone surgery was performed in all patients, where 69% had multiple BM and 46% additional extraosseous DM. Cytoreductive intraabdominal surgery has been shown to enhance OS in EC patients with stage IV disease at diagnosis, including those with BM [30], [32], [42], [72], [73]. A crucial point is the achievement of maximal cytoreduction with removal of any gross residual disease as this was shown to affect oncologic outcomes [74]. In ovarian carcinoma, potential survival benefits of optimal intraabdominal cytoreduction have been associated with the reduction of mutated and treatment-resistant tumor cells, local perfusion-enhancement and increased systemic drug-delivery, as well as reduction of carcinoma-associated metabolic effects, improving performance status [75], [76], [77]. Therefore, local surgery with the intention to reduce tumor burden in EC metastasized to bone may have favorable prognostic effects. However, metastasis-localization and spread, resectability as well as patient characteristics and performance status have to be considered and discussed within the interdisciplinary team for optimal selection of candidates for surgical resection [51], [78].

Radiotherapy in EC can be applied as external beam radiotherapy (EBRT) or intrauterine brachytherapy [47]. Bone metastases can be treated with palliative EBRT to alleviate pain [22]. In attempts to pursue complete cure or increase survival, multiple studies have recently investigated stereotactic ablative radiotherapy (SABRT), employing the precise delivery of large dose fractions especially in oligometastatic patients with various controlled primary cancers [79], [80], [81], [82], [83], [84], [85], [86], [87], [88], [89]. In the included studies of this systematic review local radiotherapy to metastatic bones was performed in two-thirds of patients irrespective of metastatic tumor burden, however no clear survival effect could be seen. This may be due to the fact that local radiotherapy is frequently used for symptom palliation in advanced settings [5], [22]. We were not able to differentially assess indications for radiotherapy in the included samples. Considering the advances using SABRT, we could not discriminate between the used methods of EBRT and the applied dosage and fractions. Previously, patients with lung oligometastasis undergoing SABRT were shown to have comparable results to surgical metastasectomy [84]. So far, the phase II randomized SABR-COMET trial (NCT01446744) showed some of the most promising clinical results. This trial included oligometastatic patients with 1–5 metastatic lesions, who were randomized to receive either standard of care palliative treatment alone or combined with SABRT. The trial included patients with BM (31% in the control arm, 35% in the treatment arm), however only two patients had gynecologic malignancies. After 5-years of follow-up, SABRT was considered safe (adverse events grade ≥ 2 in 29% of the treatment arm vs. 9% in control arm), without differences in quality of life. SABRT led to a significantly decreased progression-free survival (PFS), yet a prolonged OS [87]. Two follow-up phase III trials, namely SABR-COMET-3 (NCT03862911) including patients with 1–3 metastases, and SABR-COMET-10 (NCT03721341) including patients with 4–10 metastases are currently ongoing. Of note, patients with femur metastases are excluded from the SABR-COMET trials. A recent systematic review addressed the role of SABRT in oligometastatic gynecologic malignancies including 11.1% uterine malignancies and 15 BM. Local control rates were high between 71 and 100%, and disease progression occurred mainly outside of the SABRT field, being potential candidates for re-SABRT [81]. Although local ablative therapies have seen an increased usage in recent years especially in oligometastatic situations with a relevant number of studies reporting promising outcomes, cautious interpretation of study results is warranted. Few studies represent adequately powered randomized controlled samples, questioning the generalizability of these results and comparability to standard of care treatment [90].

### 4.2 Systemic therapy

The treatment landscape in metastatic and recurrent EC has very recently changed by the reporting of a superior PFS in two phase-III randomized controlled trials when administering immune-checkpoint inhibitors, namely dostarlimab and pembrolizumab, with a carboplatin-paclitaxel backbone compared to chemotherapy alone [17], [18]. Until then, platinum-based multiagent chemotherapy, or hormonal therapy in hormone-receptor positive, well-differentiated carcinoma were considered as first-line treatment in stage IVb EC [46], [47]. The less toxic carboplatin + paclitaxel (CT) combination was shown to be non-inferior to the TAP regimen (Doxorubicin + Paclitaxel + Cisplatin) in the GOG 209 trial [91]. Consecutive treatment lines have shown response rates < 20%, median PFS of 2–5 months, and no improvement in OS [47]. Treatment of these patients therefore represents an unmet clinical need.

In our review chemotherapy was performed frequently (median 55.5%) without numerical differences according to metastatic burden in the investigated cohorts (Table 4). Although only a small number provided detailed information on therapeutic regimens, these conferred largely to the above-mentioned recommended classic treatment approaches. Survival could be analyzed in two samples, consisting of stage IVb disease with BM at diagnosis (n = 948), as well as five samples comprising mostly recurrent cases (n = 167). Interestingly, in patients with BM at diagnosis, a clear survival benefit was noted in the investigated samples of patients undergoing chemotherapy, whilst in those samples with more recurrent disease, no survival benefit was seen. As approximately one-quarter of patients with bone recurrence had received adjuvant chemotherapy, a potential factor in these cases could be chemotherapy-resistance. However, it could also be a surrogate parameter for more frail patients with reduced performance status, and therefore not directly therapy-related. Hormone-therapy using progestins, and osteooncologic treatment approaches were performed less frequently in the included cohorts. Progestin therapy shows relatively low rates of side-effects and is therefore used as palliative treatment in advanced settings. In this review, no clear effects on survival were observed. The use of osteooncologic treatment in the investigated studies was very low, which is not consistent with international guidelines recommending these therapeutics in patients with BM, based on randomized controlled trials [22], [92], [93]. However, no explicit information on the use of zoledronic acid or denosumab in patients with BM in EC could be extracted from the randomized-controlled phase III trials by Rosen et al. investigating the efficacy and safety of zoledronic acid, nor from the trial by Henry et al. investigating the use of denosumab compared to zoledronic acid [92], [93]. Osteooncologic treatment is used to decrease the risk of SREs in patients with BM [94]. The benefit of osteooncologic treatment to relieve metastatic bone pain is considered similar to local radiotherapy [22]. Whilst we can only speculate on the reasons for the low usage of osteooncologic therapy in the investigated studies, one reason might be the covered treatment periods of 1984–2019 for the included retrospective cohort studies, and 1933–2021 for the included case studies, partially covering a period before publication of high-level evidence. Interestingly, when analyzing the included case studies by publication period, an increase in use of osteooncologic therapy could be seen from before 2000 (0%, 0/28), over 2000–2010 (11.1%, 3/27), to the period as of 2010 (23.1%, 12/52) as depicted in Table S3.

The current treatment landscape in metastatic EC is changing. Multiple treatment options have emerged since the publication of TCGA molecular characteristics one decade ago. Not only have the molecular subtypes shown both a prognostic and predictive value; current guidelines promote risk stratification in early EC and therapy decisions in recurrent and advanced EC according to molecular features [46], [47]. In the advanced setting emerging data integrate molecular changes in the treatment landscape [95], [96].

Immune-Checkpoint Inhibitors (ICI) have already changed the treatment landscape in metastatic EC. Pembrolizumab showed an ORR of 48% and a median PFS of 13.1 months in previously treated advanced dMMR EC in the Keynote-158 study [97]. Dostarlimab, an anti-programmed death 1 (PD1) antibody like pembrolizumab showed similar results, with an ORR of 42–47% in dMMR EC and a median PFS of 8.3 months [98], [99]. Based on these data, both drugs were approved as monotherapies for patients with MSI EC having progressed on first-line systemic therapy. Most recently, results from the RUBY trial (NCT03981796) investigating the combination of dostarlimab compared to placebo with a carboplatin and paclitaxel backbone after 24-months, as well as the NRG-GY018 trial (NCT03914612) investigating the combination of pembrolizumab or placebo to carboplatin-paclitaxel after 12-months have been reported. Both trials showed superior PFS in the dMMR population (61% vs. 16% after 24-months, HR 0.28, 95 %CI 0.16–0.50 in the RUBY trial; 74% vs. 38% after 12-months, HR 0.30, 95 %CI 0.19–0.48 in the NRG-GY018 trial) as well as in the mismatch-repair proficient (pMMR) population (28% vs. 19% after 24-months, HR 0.76, 95 %CI 0.59–0.98 in the RUBY trial; HR 0.54, 95 %CI 0.41–0.71 in the NRG-GY018 trial after 12-months) [17], [18]. Furthermore, investigators from the RUBY trial have also shown significant OS benefit in the overall population undergoing trial treatment in the 24-months interim analysis (71% vs. 56% after 24-months, HR 0.64, 95 %CI 0.46–0.87), which was more pronounced in the dMMR population (83% vs. 59% after 24-months, HR 0.30, 95 %CI 0.13–0.70) [17]. Therefore, the combination of ICIs with carboplatin and paclitaxel may become the new first-line treatment standard in advanced, metastatic or recurrent EC. In the phase II 111/Keynote-146 study the combination of pembrolizumab-lenvatinib was shown to be effective with an ORR of 36.2%. In dMMR patients the ORR was 63.6% [100]. Lenvatinib is a multikinase inhibitor which was primarily approved in thyroid cancer. It blocks kinase activity of VEGF, FGFR and RET. The findings of this phase II study could be confirmed irrespective of MMR status in the phase III Keynote-775/Study 209 trial, including patients with advanced, metastatic, or recurrent EC having progressed on or after platinum-based chemotherapy. Here, an improved PFS (7.2 vs. 3.8 months) and OS (18.3 vs. 11.4 months) compared to standard chemotherapy could be shown [101]. Side effects are common with ICIs, with combination regimens in the RUBY and NRG-GY018 trials showing higher rates of grade ≥ 3 adverse events (71% vs. 60% in the RUBY trial; 57% vs. 46% in the NRG-GY018 trial). Of note, the combination therapy with lenvatinib showed higher rates of adverse events compared to PD1 monotherapy (grade ≥ 3 in 88.9% with combination therapy, 12–14.6% with pembrolizumab monotherapy, 11.5–16.6% with dostarlimab monotherapy) [97], [98], [101]. In light of the above-mentioned positive studies, multiple clinical trials evaluating the role of 1st line ICI in the advanced setting are ongoing (Table 6).

**Table 6:** Phase III clinical trials of immune checkpoint inhibitors in stage IV or recurrent endometrial carcinoma [clinicaltrials.gov accessed 23rd April 2023; alphabetical order].

**Trial**	**Investigated Treatment line**	**Experimental arm**	**Control arm**	**Status**
AtTEnd NCT03603184	1st line	Atezolizumab and Carboplatin and Paclitaxel	Placebo and Carboplatin and Paclitaxel	Active, not recruiting
DOMENICA NCT05201547	1st line in dMMR	Dostarlimab	Carboplatin and Paclitaxel	Recruiting
DUO-E NCT04269200	1st line	(i) Durvalumab and standard of care chemotherapy (platinum based) followed by durvalumab and olaparib-placebo (ii) Durvalumab and standard of care chemotherapy (platinum based) followed by durvalumab and olaparib	Placebo and standard of care chemotherapy (platinum based) followed by durvalumab-placebo and olaparib-placebo	Recruiting
Keynote 775 NCT03517449	1st line	Lenvatinib and Pembrolizumab	Physician’s choice (Doxorubicin or Paclitaxel)	Active, not recruiting
LEAP-001 NCT03884101	1st line	Lenvatinib and Pembrolizumab	Carboplatin and Paclitaxel	Active, not recruiting
MK-3475-C93 NCT05173987	1st line in dMMR	Pembrolizumab	Chemotherapy (Carboplatin and Paclitaxel)	Recruiting
NRG-GY018 NCT03914612	1st line	Pembrolizumab and Carboplatin and Paclitaxel followed by Pembrolizumab	Placebo and Carboplatin and Paclitaxel followed by Placebo	Active, not recruiting
RUBY – Part 1 NCT03981796	1st line	Dostarlimab and Carboplatin and Paclitaxel followed by Dostarlimab	Placebo and Carboplatin and Paclitaxel followed by Placebo	Active, not recruiting
RUBY – Part 2 NCT03981796	1st line	Dostarlimab and Carboplatin and Paclitaxel followed by Dostarlimab and Niraparib	Placebo and Carboplatin and Paclitaxel followed by Placebo	Active, not recruiting

dMMR – mismatch repair deficient.

Treatment combinations of, for example immunotherapy and SABRT may show synergistic effects with clinical trials currently ongoing [102]. The phase II PRIMMO trial (NCT03192059) is one of the first such studies. Here, patients with advanced and recurrent endometrial carcinoma, uterine sarcoma, and cervical cancer who were treated with an immunomodulatory cocktail, 3-weekly pembrolizumab and SABRT (3x8Gy in 48-h intervals) were included. Recently published results showed an unmet primary endpoint, with an immune-related overall response rate of 12% in uterine cancer and 11.1% in cervical cancer [89].

Patients with p53abn serous-like EC harbor the worst prognosis, however analyses from the PORTEC-3 trial have shown improved 5-year recurrence-free survival for patients who underwent combined adjuvant chemoradiotherapy compared to radiotherapy alone [95]. Furthermore, these carcinomas are associated with homologous recombination deficiency (HRD), making them prone to treatment with PARP-inhibitors (PARPi). Currently, two trials are investigating PARPi in advanced or recurrent EC. In the second part of the above-mentioned RUBY trial, patients are randomized to undergo maintenance-therapy with dostarlimab and the PARPi niraparib or placebo. The DUO-E trial (NCT04269200) is currently recruiting patients with newly diagnosed advanced or recurrent EC as outlined in Table 6. Another well-established predictive factor in other tumor entities such as breast cancer is HER2 overexpression, which is present in almost 25% of p53abn EC. Anti-Her2 monotherapy did not show improved clinical outcomes, whilst the combination of trastuzumab with platinum-based chemotherapy found a prolonged PFS and OS in patients with stage III to IV EC [103]. Currently, trials with the antibody-drug-conjugate trastuzumab-deruxtecan are ongoing including patients with advanced or metastatic EC [104], [105]. Moreover, wee1-inhibitors, a G2 checkpoint regulating protein kinase have shown preclinical and clinical activity in EC [106], [107], [108]. The Phase IIb ADAGIO trial (NCT04590248) has been designed to evaluate the efficacy of adavosertib in women with recurrent or persistent uterine serous carcinoma, with much awaited results [109]. Other targeted therapies are also continuously being investigated, as done in e.g., the GOG 86P trial, using CT + Bevacizumab to the GOG 209 sample not showing enhanced survival [110].

Moreover, there is a high rate of estrogen receptor (ER) expressing EC. Progestin derivatives were a mainstay of therapy in patients with slowly progressing, low-grade, low volume ER-positive stage IV disease, showing response rates of 25% and clinical benefit in 37% [111], [112]. Response rates with other anti-hormonal agents are 8–9% for aromatase inhibitors, 10–53% for tamoxifen, and 9–31% for selective estrogen receptors modulators and downregulators such as fulvestrant in advanced and recurrent EC. Combination therapies of tamoxifen and progestin result in a response in 19–58% [111]. A low response rate of 7% with anastrozole was confirmed in the phase II PARAGON trial. However, a clinical benefit could be observed in 44% of patients with a maintained QoL [113]. Therapy combinations have also been assessed using mTor inhibitors and endocrine agents. The phase I/II randomized VICTORIA trial showed an enhanced response rate of 25% when combining vistusertib with anastrozole with a prolonged PFS and increased yet manageable toxicities [114]. Letrozole in combination with the cyclin-dependent kinase 4/6 (CDK4/6) inhibitor palbociclib has been assessed in the phase II NSGO-PALEO/ENGOT-EN3 trial. The combination treatment showed a significantly prolonged PFS and disease control rate with more grade 3/4 toxicities compared to letrozole and placebo [115]. Other clinical trials are currently investigating the combination of abemaciclib and letrozole (NCT03675893), narazaciclib plus letrozole (NCT05705505), lerociclib and letrozole (NCT05712941) and abemaciclib monotherapy (NCT04469764) [116], [117], [118], [119].

### 4.3 Practical implications

Based on stratification factors outlined in the beginning of the discussion, available guidelines [22], [46], [47], and the presented evidence we propose the following treatment recommendations for certain clinical scenarios of BM in EC. This should always be discussed within the multidisciplinary team and with the respective patient to offer an individualized treatment plan. If possible, trial enrollment should also be considered in these patients.

In patients presenting with an oligometastatic situation including isolated BM being treatment-naïve or with a chemotherapy free period of at least 6-months, systemic chemotherapy including carboplatin and a taxol should be used. Based on the recently published results of the RUBY and NRG-GY018 trials, adding dostarlimab or pembrolizumab may be considered, especially in patients with dMMR EC. In case of progression on chemotherapy, both PD1-antibodies are approved as monotherapies, with Pembrolizumab-Lenvatinib representing a treatment-alternative, again especially in patients with dMMR EC. Patients who have not received trastuzumab in Her2-positive disease, chemotherapy in combination with trastuzumab represents an option in serous carcinoma or carcinosarcoma. Patients with a hormone receptor positive EC, being ineligible for chemotherapy may receive anti-hormonal therapy. Osteooncologic therapy should be included in all patients with a life expectancy of ≥ 3 months. Radical local ablative therapies may be considered in this scenario, with SABRT currently showing more robust evidence compared to local bone metastasectomy, although both have shown improved oncologic outcomes. In general, patients should present with an ECOG score of 0–2 or a Karnofsky-Index of 100–60 to be offered the proposed local therapy regimens with curative intent. In cases of primary diagnosis of EC with BM, surgical approaches should include total hysterectomy and bilateral salpingo-oophorectomy as well as maximal intraabdominal cytoreduction if feasible. Local bone surgery should be considered to stabilize pathologic fractures, prevent impending fractures and neural compression. Treatment sequence may be individualized, using local ablative strategies first if maximal cytoreduction is feasible followed by subsequent systemic therapy. On the converse, systemic therapy should be considered first line in patients where optimal cytoreduction is deemed unlikely and no local treatment pressure exists. Local therapies should be re-evaluated on an individual level according to treatment response.

Systemic therapy-approaches in patients with widespread metastatic disease and disseminated BM remain unchanged to the above-mentioned scenario. Local ablative therapies should be considered as palliative treatment for symptom control, at least until results from SABR-COMET 10 are reported.

### 4.4 Future research directions

Our results comprehensively review available evidence regarding therapeutic approaches and survival impact in patients with BM in EC, which is currently an under-investigated research area. The role of local ablative therapies in DM lesions is currently being addressed in clinical trials. Local cytoreductive surgery seems to have beneficial outcomes for selected patients, whereas SABRT also shows promising first results. However, both strategies are yet to be validated especially in BM. Furthermore, as mentioned in the discussion a lack of knowledge exists on SREs in patients with BM in EC and their therapeutic approaches. The landscape of systemic therapies in advanced and recurrent EC is fast evolving, with a multitude of ongoing phase-III clinical trials offering multiple promising approaches also for patients with BM in EC. Very recently, Pradat et al. published their resource paper on “Integrative pan-cancer genomic and transcriptomic analyses of refractory metastatic cancer” including six samples of patients with primary uterine cancer. Herein, the authors showed, a significant increase in somatic mutations and tumor mutational burden in over 1000 refractory carcinomas compared to the TCGA cohort. Furthermore, current standard resistance biomarkers were present in only a minority of analyzed samples, highlighting the need for translational cancer analyses of metastatic patients [120]. Whilst the molecular profiling of non-metastatic EC has shown a relevant prognostic, predictive and therapeutic impact, characterization of metastatic EC potentially harbors so far underused individualized therapeutic opportunities and, in our opinion, represents the most important field of future research, especially following the diagnosis of metastatic dMMR EC and p53mut. The latter potentially also representing a subtype, which harbors an increased risk of BM [34]. In our analysis unfortunately only nine cases with available molecular information could be identified in the reviewed studies, with one being of NSMP, and eight presenting MSI. Such information investigated and integrated in advanced settings potentially confers to distinct prognosis and treatment options in metastatic disease as well.

### 4.5 Study limitations

Limitations of this analysis include the heterogeneity of the investigated study populations, the largely small sample sizes of the individual studies, as well as the lack of controlled intervention studies. The retrospective nature of the investigated samples makes them prone to selection bias. We did include case studies in this review, as we found relevant information on patient characteristics, treatment approaches, and survival could be extracted from those, especially in light of the limited sample numbers in other studies, and the abundance of literature available in this format. However, case studies show an inherent selection-bias, as only “special” cases may be published, potentially not being representative of the general sample. On the other hand, as BM in EC are per se special, we do believe, that this risk may be limited. Furthermore, we followed recently established protocols to comprehensively include case studies in systematic reviews [40]. Finally, statistically only descriptive analyses and univariate HR were reported without correction for possible influence factors on survival and without the possibility to assess other cancer-related outcomes due to heterogeneous reporting. Therefore, we summarized and described effect estimates without being able to account for differences in the relative study-sizes. We abstained from comparative statistical analysis and *meta*-analysis due to relevant clinical, methodological, and statistical sample heterogeneity.

## 5 Conclusions

This is the first systematic review investigating detailed frequencies of therapeutic modalities in patients with BM in EC and their impact on prognosis, comprehensively summarizing currently available evidence, and potentially guiding future treatment as well as research approaches. Currently, treatment approaches are heterogeneous without clear evidence for optimal oncologic management for patients with BM in EC.

## Institutional review board Statement

An institutional review board approval is not applicable because this study is based exclusively on published literature.

## Informed consent Statement

Not applicable.

## Funding

This research did not receive any specific grant from funding agencies in the public, commercial, or not-for-profit sectors.

## CRediT authorship contribution statement

**Martin Heidinger:** Conceptualization, Methodology, Formal analysis, Investigation, Visualization, Data curation, Writing – original draft, Writing – review & editing. **Elisa Simonnet:** Methodology, Investigation, Data curation, Writing – review & editing. **Li Mei Koh:** Investigation, Writing – original draft, Writing – review & editing. **Brigitte Frey Tirri:** Conceptualization, Supervision, Writing – review & editing. **Marcus Vetter:** Conceptualization, Methodology, Writing – review & editing, Supervision.

## Declaration of Competing Interest

The authors declare that they have no known competing financial interests or personal relationships that could have appeared to influence the work reported in this paper.
