# Supplementary material for: Therapeutic approaches in patients with bone metastasis due to endometrial carcinoma – A systematic review
Source: J Bone Oncol. 2023 May 15;41:100485. doi: 10.1016/j.jbo.2023.100485 (PMC10213377; doi:10.1016/j.jbo.2023.100485)
Supplement: Supplementary data 1 [file mmc1.docx]

# Supplementary Materials

Therapeutic approaches in patients with bone metastasis due to endometrial carcinoma – a systematic review

Martin Heidinger ^1,2,3,*^, Elisa Simonnet ^1^, Li Mei Koh ^1^, Brigitte Frey Tirri ^1^, Marcus Vetter ^2,4^

^1^ Women’s Clinic, Cantonal Hospital Baselland, Rheinstrasse 26, 4410 Liestal, Switzerland

^2^ University of Basel, Petersplatz 1, 4001 Basel, Switzerland

^3^ Breast Center, University Hospital Basel, Spitalstrasse 21, 4031 Basel, Switzerland

^4^ Medical Oncology, Cantonal Hospital Baselland, Medical University Clinic, Muehlemattstrasse 13, 4410 Liestal, Switzerland

***** Corresponding author: Dr. med. univ. Martin Heidinger

martin.heidinger@usb.ch

University Hospital Basel

Spitalstrasse 21, 4031 Basel

Switzerland

### Appendix A - Search Strategies

**PubMed - run 27/03/2022**

((endometrial neoplasms[mesh] OR (endometrium[tiab] OR endometrial[tiab] OR uterine mucosa[tiab] OR uterus mucosa[tiab]) AND (neoplas*[tiab] OR tumor*[tiab] OR tumour*[tiab] OR cancer*[tiab] OR carcinoma*[tiab] OR malignan*[tiab]))) AND (bone neoplasms[mesh] OR (("bone and bones"[mesh] OR bone*[tiab] OR skelet*[tiab] OR osseous[tiab] OR osteoblastic[tiab] OR osteoplastic[tiab]) AND ("Carcinoma, Endometrioid/secondary"[Mesh] OR neoplasm metastasis[mesh:noexp] OR metasta*[tiab] OR tumor migration[tiab] OR tumour migration[tiab] OR invasion[tiab] OR disseminat*[tiab] OR spread[tiab] OR secondary cancer*[tiab] OR secondary carcinoma*[tiab])))

**MEDLINE via Ovid - run 27/03/2022**

((exp "endometrial neoplasms"/ OR (endometrium.tw. OR endometrial.tw. OR "uterine mucosa".tw. OR "uterus mucosa".tw.) AND (neoplas*.tw. OR tumor*.tw. OR tumour*.tw. OR cancer*.tw. OR carcinoma*.tw. OR malignan*.tw.))) AND (exp "bone neoplasms"/ OR ((exp "bone and bones"/ OR bone*.tw. OR skelet*.tw. OR osseous.tw. OR osteoblastic.tw. OR osteoplastic.tw.) AND (exp "Carcinoma, Endometrioid/secondary"/ OR "neoplasm metastasis"/ OR metasta*.tw. OR "tumor migration".tw. OR "tumour migration".tw. OR invasion.tw. OR disseminat*.tw. OR spread.tw. OR "secondary cancer*".tw. OR "secondary carcinoma*".tw.)))

**Embase via Elsevier - run 27/03/2022**

(('endometrium cancer'/exp OR (endometrium:ti,ab OR endometrial:ti,ab OR 'uterine mucosa':ti,ab OR 'uterus mucosa':ti,ab) AND (neoplas*:ti,ab OR tumor*:ti,ab OR tumour*:ti,ab OR cancer*:ti,ab OR carcinoma*:ti,ab OR malignan*:ti,ab)) AND ('bone metastasis'/exp OR (('bone'/exp OR bone*:ti,ab OR skelet*:ti,ab OR osseous:ti,ab OR osteoblastic:ti,ab OR osteoplastic:ti,ab) AND (metasta*:ti,ab OR 'tumor migration':ti,ab OR 'tumour migration':ti,ab OR invasion:ti,ab OR disseminat*:ti,ab OR spread:ti,ab OR 'secondary cancer*':ti,ab OR 'secondary carcinoma*':ti,ab))))

**ClinicalTrials.gov - run 27/03/2022**
("Endometrial Cancer" AND "Bone metastasis" AND "Survival" AND ("Phase 3" OR "Phase 4" OR "Not Applicable"))

### Appendix B – Excluded full-text articles

Language

1. Witczak Ploch E. A case of successful hormonal treatment of a patient with endometrial carcinoma and extensive metastases to pelvic bones. Nowotwory 1983; 33: 139–141.

2. Mitomo O. Metastatic malignancy to the maxilla; Report of two cases. Japanese J Clin Radiol 1985; 30: 113–116.

3. Zhao Y. Clinical analysis of eight cases of bone metastasis of uterine carcinomas. Zhonghua Fu Chan Ke Za Zhi 2006; 41: 822–825.

4. Gawlak M. Cutaneous and bony metastases after treatment of endometrial cancer - Case report and review of the literature. Curr Gynecol Oncol 2012; 10: 244–252.

5. Jiang F. Clinicopathological features and prognosis of patients in endometrial cancer with bone metastases. Zhonghua Fu Chan Ke Za Zhi 2019; 54: 452–457.

No information on bone metastasis

1. Ballon SC. Pulmonary metastases of endometrial carcinoma. Gynecol Oncol 1979; 7: 56–65.

2. Vardi JR. The value of exploratory laparotomy in patients with endometrial carcinoma  according to the new International Federation of Gynecology and Obstetrics staging. Obstet Gynecol 1992; 80: 204–208.

3. Yoney A. Retrospective analysis of 105 cases with uterine sarcoma. Bull Cancer 2008; 95: E10-7.

4. Tanioka M. Clinical characteristics and outcomes of women with stage IV endometrial cancer. Med Oncol 2010; 27: 1371–1377.

5. Di Legge A. Phase 2 trial of nonpegylated doxorubicin (Myocet) as second-line treatment in advanced or recurrent endometrial cancer. Int J Gynecol Cancer 2011; 21: 1446–1451.

6. Sorbe B. A population-based series of uterine carcinosarcomas with long-term follow-up. Acta Oncol (Madr) 2013; 52: 759–766.

7. Tirumani SH. Metastatic pattern of uterine leiomyosarcoma: Retrospective analysis of the predictors and outcome in 113 patients. J Gynecol Oncol 2014; 25: 306–312.

8. Shimamoto K. Prognostic significance of the treatment-free interval in patients with recurrent endometrial cancer. Eur J Obstet Gynecol Reprod Biol 2014; 175: 92–96.

9. Bartosch C. Distant Metastases in Uterine Leiomyosarcomas. Int J Gynecol Pathol 2017; 36: 31–41.

10. Bricou A. A Proposal for a Classification for Recurrent Endometrial Cancer: Analysis of a French Multicenter Database from the FRANCOGYN Study Group. Int J Gynecol Cancer 2018; 28: 1278–1284.

11. Cuccia F. The use of SBRT in the management of oligometastatic gynecological cancer: report of promising results in terms of tolerability and clinical outcomes. J Cancer Res Clin Oncol 2021; 147: 3613–3618.

No information on endometrial carcinoma

1. Baron MG. Bone metastases as the first manifestation of a tumour. Int Orthop 1991; 15: 373–376.

2. Bollen L. Predictive value of six prognostic scoring systems for spinal bone metastases. Spine (Phila Pa 1976) 2016; 41: E155–E162.

3. Yuan L. Clinical analysis of surgical treatment of long bone metastases. Natl Med J China 2016; 96: 1500–1504.

4. Bahrabadi M. Foot metastasis: Review of 38 cases. Arch Bone Jt Surg 2021; 9: 122–125.

No survival information

1. Rouchy R. Osseous metastasis revealing endometrial cancer. Bull Fed Soc Gynecol Obstet Lang Fr 1967; 19: 352–353.

2. Gaujoux J. Bone metastasis revealing a cancer of the endometrium. Mars Med 1972; 109: 437–440.

3. Janis LR. Metastatic adenocarcinoma of the calcaneus: case report. J Foot Surg 1976; 15: 28–32.

4. Brufman G. Metastatic bone involvement in gynecological malignancies. Radiol Clin 1978; 47: 456–463.

5. Zorzi R. Metastasis of endometrial carcinoma to the tarsus. Chir Organi Mov 1982; 68: 727–730.

6. Scott A. Ethmoid metastasis of endometrial carcinoma causing mucocoele of maxillary  antrum. J Laryngol Otol 1998; 112: 283–285.

7. Galen DM. Mandibular metastasis of endometrial carcinoma diagnosed via a dental radiograph. J Am Dent Assoc 1998; 129: 1595–1598.

8. Landoni F. Endometrial carcinoma bone metastases in unusual sites. Gynecol Oncol 2006; 102: 411.

9. Walrath JD. Metastatic endometrial carcinoma resulting in orbital apex compression. Ophthal Plast Reconstr Surg 2007; 23: 250–251.

10. Farooq MU. Intracranial and scalp metastasis of endometrial carcinoma. Med Sci Monit 2008; 14: CS87–CS88.

11. Ho L. Intramuscular metastasis of endometrial carcinoma on FDG PET/CT. Clin Nucl Med 2010; 35: 607–609.

12. Bryant CS. Sacral metastases in an endometrial cancer patient after treatment with sequential chemo-radiotherapy: A case report. Arch Oncol 2010; 18: 38–39.

13. Huang MI. Endometrial stromal sarcoma metastasis to the lumbar spine and sphenoid bone. Rare Tumors 2011; 3: e27.

14. Bayraktutan U. Endometrial cancer metastasis mimicking spondylodiscitis and psoas abscess. Spine J 2016; 16: e9–e10.

15. Khoo ACH. Recurrent Endometrioid Adenocarcinoma of Endometrium With Bone Metastases. Clin Nucl Med 2017; 42: 635–636.

16. Kawashima I. Bone marrow invasion of small cell neuroendocrine carcinoma of the endometrium: A diagnostic pitfall mimicking a haematological malignancy. Intern Med 2019; 58: 2561–2568.

17. Bidkar VC. Neuroendocrine Carcinoma of Female Genital Tract: Series of Nine Cases. Indian J Gynecol Oncol; 19. Epub ahead of print 2021. DOI: 10.1007/s40944-020-00480-x.

18. Wong B. Recurrent endometrial carcinoma metastasis to the temporal bone and skull base. Ann R Coll Surg Engl 2021; 103: e338–e340.

19. Levinson JS. Endometrial Sarcoma Metastasis to the Pterygopalatine Fossa: A Case Report and Review of the Literature. Ear, Nose Throat J. Epub ahead of print 2021. DOI: 10.1177/0145561320983943.

20. Birge Ö. A case of cranial bone metastasis after fertility-sparing approach in endometrial cancer. J Obstet Gynaecol (Lahore). Epub ahead of print 2022. DOI: 10.1080/01443615.2021.2012440.

No treatment information

1. Orlian AI. Metastatic endometrial carcinoma to the maxilla. N Y State Dent J 1978; 44: 188–189.

2. Sharma DN. Medical disorders associated with endometrial carcinoma. J Assoc Physicians India 2001; 49: 630–633.

3. Takeshita S. A prediction model of survival for patients with bone metastasis from uterine corpus cancer. Jpn J Clin Oncol 2016; 46: 973–978.

4. Li H. Prognostic value of different metastatic sites for patients with FIGO stage IVB endometrial cancer after surgery: A SEER database analysis. J Surg Oncol 2020; 122: 941–948.

5. Mao W. Clinicopathological study of organ metastasis in endometrial cancer. *Futur Oncol* 2020; 16: 525–540.

Not retrievable upon multiple contacting

1. Rosa MA. Endometrial carcinoma and bone metastases: Diagnostic and clinical characteristics. Minerva Ortop e Traumatol 2001; 52: 275–278.

2. Woo A. Solitary bone metastatic lesion to the cuboid in endometrial cancer and a review of the literature. J Pain Manag 2015; 8: 261–264.

3. Yee C. Tibial metastasis as a presenting symptom of endometrial adenocarcinoma: A case report. J Pain Manag 2018; 11: 193–197.

Orbital metastasis

1. Char DH. Cystic orbital metastasis from endometrial carcinoma. Orbit 2007; 26: 75–77.

Skin metastasis

1. Khurelbaatar T. Scalp metastasis from endometrial carcinoma: a case report and literature review. J Obstet Gynaecol (Lahore) 2018; 38: 1181–1182.

More recent report on the same patient available

1. Petru E. Solitary metastasis in the tarsus preceding the diagnosis of primary endometrial cancer - A case report. Eur J Gynaecol Oncol 1995; 16: 387–391.

(More recent publication: Benedicic C. Tarsal metastasis as the primary manifestation of endometrial cancer. A case report. Geburtshilfe Frauenheilkd 2002; 62: 179–180.)

### Appendix C – Included full-text articles

Cohort studies

1. Kehoe SM. Clinicopathologic features of bone metastases and outcomes in patients with primary endometrial cancer. Gynecol Oncol 2010; 117: 229–233.

2. Blecharz P. Hematogenous metastases in patients with Stage i or II endometrial carcinoma. Strahlentherapie und Onkol 2011; 187: 806–811.

3. Uccella S. Bone metastases in endometrial cancer: report on 19 patients and review of the  medical literature. Gynecol Oncol 2013; 130: 474–482.

4. Yoon A. Bone metastasis in primary endometrial carcinoma features, outcomes, and predictors. Int J Gynecol Cancer 2014; 24: 107–112.

5. Kimyon G. Bone recurrence rarely seen in endometrial cancer and review of the literature. J Obstet Gynaecol Res 2016; 42: 602–611.

6. Ouldamer L. Incidence, patterns and prognosis of first distant recurrence after surgically treated early stage endometrial cancer: Results from the multicentre FRANCOGYN study group. Eur J Surg Oncol 2019; 45: 672–678.

7. Guo J. The Clinical Characteristics of Endometrial Cancer With Extraperitoneal Metastasis and the Value of Surgery in Treatment. Technol Cancer Res Treat 2020; 19: 1533033820945784.

8. McEachron J. A clinicopathologic study of endometrial cancer metastatic to bone: Identification  of microsatellite instability improves treatment strategies. Gynecologic oncology reports 2020; 32: 100549.

9. Liu Y. Prognostic value of distant metastatic sites in stage IV endometrial cancer: A SEER database study of 2948 women. Int J Gynecol Obstet 2020; 149: 16–23.

10. Yang F. Diagnostic and prognostic factors, and two nomograms for endometrial cancer patients with bone metastasis: A large cohort retrospective study. Medicine (Baltimore) 2021; 100: e27185.

11. Hu H. Clinicopathological Characteristics and Prognosis in Endometrial Cancer With Bone Metastasis: A SEER-Based Study of 584 Women. Front Oncol;

12. Wang J. Bone Metastases of Endometrial Carcinoma Treated by Surgery: A Report on 13  Patients and a Review of the Medical Literature. Int J Environ Res Public Health; 19. Epub ahead of print June 2022. DOI: 10.3390/ijerph19116823.

Case studies

1. Finn WF. Time, site, and treatment of recurrences of endometrial carcinoma. *Am J Obstet Gynecol* 1950; 60: 773–782.

2. Milch H. Endometrial carcinoma metastatic to the ischial tuberosity. *AMA Arch Surg* 1953; 66: 686–692.

3. Vanecko RM. Metastasis to the fibula from endometrial carcinoma. Report of 2 cases. *Obstet Gynecol* 1967; 29: 803–805.

4. Ravault P. Métastase isolée du scaphoĭde tarsien au cours d’un cancer du corps utérin” [Isolated metastasis of the tarsal scaphoid bone in the course of cancer of the uterine body]. *Rev Rhum Mal Osteoartic* 1967; 34: 650–654.

5. Gelberman RH. Bone metastasis from carcinoma of the uterus. A case report. *CLINORTHOP* 1975; No. 106: 148–150.

6. Boidi-Trotti A. Some cases of bone metastasis in uterine cancer. *Minerva Ginecol* 1978; 30: 527–530.

7. Beller U. Early osseous metastasis of stage 1 endometrial carcinoma: Report of a case. *Gynecol Oncol* 1982; 14: 141–146.

8. Debois JM. Endometrial Adenocarcinoma Metastatic to the Scalp: Report of Two Cases. *Arch Dermatol* 1982; 118: 42–43.

9. Le Loët X. A case of a single tibial metastasis disclosing cancer of the endometrium. *Rev Rhum Mal Osteoartic* 1983; 50: 246–247.

10. Onuba O. Pathological fracture of right tibia, an unusual presentation of endometrial carcinoma: A case report. *Injury* 1983; 14: 541–545.

11. Aalders JG. Stage VI endometrial carcinoma: A clinical and histopathological study of 83 patients. *Gynecol Oncol* 1984; 17: 75–84.

12. Kapp DS. Cauda equina compression secondary to metastatic carcinoma of the uterine corpus: Preservation of neurologic function and long-term survival following surgical decompression and radiation therapy. *Gynecol Oncol* 1985; 20: 209–218.

13. Lieschke GJ. Endometrial adenocarcinoma presenting as pituitary apoplexy. *Aust N Z J Med* 1990; 20: 81–84.

14. Maxymiw WG. Metastatic endometrial carcinoma to the mandible: a case report. *J oral Maxillofac Surg Off J Am Assoc Oral Maxillofac Surg* 1991; 49: 78–80.

15. Litton GJ. Isolated calcaneal metastasis in a patient with endometrial adenocarcinoma. *Cancer* 1991; 67: 1979–1983.

16. Nishida Y. Metastatic calcaneal adenocarcinoma in a patient with uterine carcinoma. *International journal of gynaecology and obstetrics: the official organ of the International Federation of Gynaecology and Obstetrics* 1994; 45: 287–288.

17. Cooper JK. Endometrial adenocarcinoma presenting as an isolated calcaneal metastasis. A rare entity with good prognosis. *Cancer* 1994; 73: 2779–2781.

18. Schöls WA. Recurrent endometrial adenocarcinoma presenting as a solitary humeral metastasis. *Gynecol Oncol* 1995; 59: 148–150.

19. Giardina VN. Metastatic endometrial adenocarcinoma to the skin of a toe. *Am J Dermatopathol* 1996; 18: 94–98.

20. Clarke SJ. Metastatic endometrial carcinoma of the foot. A case report. *J Am Podiatr Med Assoc* 1996; 86: 331–333.

21. Kushner DM. Endometrial adenocarcinoma metastatic to the scalp: case report and literature review. *Gynecol Oncol* 1997; 65: 530–533.

22. Malicky ES. Endometrial carcinoma presenting with an isolated osseous metastasis: A case report and review of the literature. *Eur J Gynaecol Oncol* 1997; 18: 492–494.

23. Armentano G. Untreated endometrial adenocarcinoma: A case report. *Eur J Gynaecol Oncol* 1997; 18: 144–145.

24. Fukunaga M. Pelvic bone involvement in low-grade endometrial stromal sarcoma with ovarian sex cord-like differentiation. *Histopathology* 1996; 29: 391–393.

25. Dosoretz DE. Mandibular metastasis in a patient with endometrial cancer. *Gynecol Oncol* 1999; 72: 243–245.

26. Rocha WC. Endometrial carcinoma metastatic to the mandible: A case report. *J Oral Maxillofac Surg* 2000; 58: 914–916.

27. Sahinler I. Endometrial carcinoma and an unusual presentation of bone metastasis: A case report. *Gynecol Oncol* 2001; 82: 216–218.

28. Mustafa MS. Scalp and cranial bone metastasis of endometrial carcinoma: A case report and literature review. *Gynecol Oncol* 2001; 81: 105–109.

29. Benedicic C. Tarsal metastasis as the primary manifestation of endometrial cancer. A case report. *Geburtshilfe Frauenheilkd* 2002; 62: 179–180.

30. Manolitsas TP. Pain in the foot: Calcaneal metastasis as the presenting feature of endometrial cancer. *Obstet Gynecol* 2002; 100: 1067–1069.

31. Neto AG. Endometrial endometrioid adenocarcinoma in a premenopausal woman presenting with metastasis to bone: a case report and review of the literature. *Int J Gynecol Pathol Off J Int Soc Gynecol Pathol* 2002; 21: 281–284.

32. Dursun P. Bilateral bone metastasis in endometrial adenocarcinoma. *Lancet Oncol* 2003; 4: 547.

33. Ali ZA. Endometrial cancer metastasis presenting as a grossly swollen toe. *Int J Gynecol Cancer* 2003; 13: 909–911.

34. Arnold J. Prolonged survival time following initial presentation with bony metastasis in stage IVb endometrial carcinoma. *Aust New Zeal J Obstet Gynaecol* 2003; 43: 239–240.

35. Ilvan S. Endometrial clear cell carcinoma metastatic to the paranasal sinuses: a case report and review of the literature. *Gynecol Oncol* 2004; 94: 232–234.

36. Kim ES. Cutaneous metastasis of uterine papillary serous carcinoma. *Am J Dermatopathol* 2005; 27: 436–438.

37. Amiot RA. Endometrial carcinoma metastasis to the distal phalanx of the hallux: A case report. *J Foot Ankle Surg* 2005; 44: 462–465.

38. Dursun P. Skeletal carcinomatosis in endometrial clear cell carcinoma at initial presentation: A case report. *Int J Gynecol Cancer* 2006; 16: 891–895.

39. Loizzi V. Two cases of endometrial cancer diagnosis associated with bone metastasis. *Gynecol Obstet Invest* 2006; 61: 49–52.

40. Osanai T. Long-term prevention of skeletal complications by pamidronate in a patient with bone metastasis from endometrial carcinoma: A case report. *Gynecol Oncol* 2006; 100: 195–197.

41. Giannakopoulos CK. Bone metastasis as a presenting feature of endometrial adenocarcinoma: Case report and literature review. *Eur J Gynaecol Oncol* 2006; 27: 95–97.

42. Al-Salam S. Low-grade endometrial stromal sarcoma with sex cord-like differentiation metastatic to the thoracic spines. *APMIS* 2006; 114: 651–655.

43. Uharček P. Endometrial adenocarcinoma presenting with an osseous metastasis. *Gynecol Obstet Invest* 2006; 61: 200–202.

44. Haraguchi S. Resection of sternal metastasis from endometrial carcinoma followed by reconstruction with sandwiched Marlex and stainless steel mesh: Report of a case. *Surg Today* 2006; 36: 184–186.

45. Frankel BM. Segmental polymethylmethacrylate-augmented pedicle screw fixation in patients with bone softening caused by osteoporosis and metastatic tumor involvement: A clinical evaluation. *Neurosurgery* 2007; 61: 531–537.

46. Ishibashi M. Endometrial carcinosarcoma presenting as a tibial metastasis. *Int J Clin Oncol* 2007; 12: 305–308.

47. Kaya A. Solitary bone metastasis in the tibia as a presenting sign of endometrial adenocarcinoma: a case report and the review of the literature. *Clin Exp Metastasis* 2007; 24: 87–92.

48. Albareda J. Sacral metastasis in a patient with endometrial cancer: Case report and review of the literature. *Gynecol Oncol* 2008; 111: 583–588.

49. Qin Y. Bilateral femur metastasis In endometrial adenocarcinoma. *Saudi Med J* 2008; 29: 766–769.

50. Srikantia N. Endometrioid endometrial adenocarcinoma in a premenopausal woman with multiple organ metastases. *Indian J Med Paediatr Oncol* 2009; 30: 80–83.

51. Pakos EE. Solitary tibial osteolytic lesion. *Case reports in medicine* 2009; 2009: 352085.

52. Artioli G. Rare presentation of endometrial carcinoma with singular bone metastasis. *Eur J Cancer Care (Engl)* 2010; 19: 694–698.

53. Oaknin A. Muscle metastasis of low-grade endometrial carcinoma seven years after diagnosis: a case report. *Eur J Gynaecol Oncol* 2010; 31: 114–116.

54. Shigemitsu A. Endometrial cancer diagnosed by the presence of bone metastasis and treated with zoledronic acid: A case report and review of the literature. *Case Rep Oncol* 2010; 3: 471–476.

55. Chan V. Malignant Invasion of Sternotomy Incision After Cardiac Operation. *Ann Thorac Surg* 2010; 89: 1295–1296.

56. Turner S. Endometrial carcinoma diagnosed by scapular biopsy: A case report. *Gynecol Oncol case reports* 2011; 1: 10–11.

57. Kurt M. Isolated bone metastases in early stage endometrial adenocarcinoma: A case report and review of the literature. *UHOD - Uluslararasi Hematol Derg* 2011; 21: 52–56.

58. Gottwald L. An extremely rare presentation of relapse in endometrioid endometrial adenocarcinoma: Isolated metastases to the tibia and humerus. Case report and review of the literature. *Eur J Gynaecol Oncol* 2011; 32: 547–550.

59. Pieters RS. The rare presentation of sinus tarsi syndrome secondary to metastasis in a patient with endometrial carcinoma. *Radiol Case Reports* 2011; 6: 414.

60. Brown JV 3rd. Spinal epidural metastasis in an endometrial carcinoma patient. *Gynecol Oncol case reports* 2011; 2: 20–22.

61. Batista LM. Spinal metastasis of endometrial stromal sarcoma: Clinicopathological features and management. *Surg Oncol* 2011; 20: e78–e83.

62. Jiang G-Q. Clinicopathological features and treatment of extremity bone metastasis in patients with endometrial carcinoma: A case report and review. *Chin Med J (Engl)* 2011; 124: 622–626.

63. Gottwald L. Isolated metastasis to the foot as an extremely rare presenting feature of primary endometrial cancer. Case report and review of the literature. *Arch Med Sci* 2012; 8: 172–174.

64. Tang Y-H. Limb salvage treatment in a 25-year-old woman with stage IVB endometrial cancer presenting with hip bone metastasis. *Taiwan J Obstet Gynecol* 2012; 51: 465–468.

65. Chen Y-C. Minimally invasive strategy for gynecologic cancer with solitary periacetabular metastasis. *Formos J Surg* 2012; 45: 127–131.

66. Vizzielli G. External hemipelvectomy as treatment for solitary coxofemoral metastasis from endometrial carcinoma: Case report and review of the literature. *J Obstet Gynaecol Res* 2012; 38: 892–898.

67. Chen CY. Successful treatment of isolated fibular bone metastasis in a uterine endometrial cancer of clear cell carcinoma. *Eur J Gynaecol Oncol* 2013; 34: 347–349.

68. Nguyen M-LT. Grade 1 Endometrioid Endometrial Carcinoma Presenting with Pelvic Bone Metastasis: A Case Report and Review of the Literature. *Case Rep Obstet Gynecol* 2013; 2013: 1–3.

69. Myriokefalitaki E. Primary bone metastasis as initial presentation of endometrial cancer (stage IVb). *Arch Gynecol Obstet* 2013; 288: 739–746.

70. Gonzalez-Benitez C. Scalp and cranial vault fulminant relapse from an endometrial carcinoma. *J Obstet Gynaecol* 2014; 34: 744.

71. Cecchi PC. Calvarial metastasis from endometrial carcinoma: Case report and review of the literature. *Asian journal of neurosurgery* 2014; 9: 242.

72. Luedke C. Metastatic endometrial carcinoma invading bilateral total knee arthroplasties. *Arthroplasty today* 2015; 1: 31–35.

73. Longo R. An unusual solitary metatarsal metastasis from an endometrioid endometrial adenocarcinoma. *Am J Case Rep* 2015; 16: 473–477.

74. Daga D. Bone metastasis in the tibia-fibula as a presenting sign of endometrial adenocarcinoma: A case report and review of the literature. *Saudi J Heal Sci* 2015; 4: 71.

75. Ghosh S. Osseous metastases in gynaecological epithelial malignancies: A retrospective institutional study and review of literature. *J Clin Diagnostic Res* 2015; 9: XC10–XC13.

76. Boukhar S. Tibial bone metastasis as an initial presentation of endometrial carcinoma diagnosed by fine-needle aspiration cytology: A case report and review of the literature. *Cytojournal*; 12. Epub ahead of print 2015. DOI: 10.4103/1742-6413.157507.

77. Chow LTC. Delayed osseous metastasis from low-grade endometrial stromal sarcoma: Uncommon occurrence deserving recognition. *J Obstet Gynaecol Res* 2015; 41: 1669–1675.

78. Lunardi P. Bilateral femur metastases in low-grade endometrial carcinoma. *Clin Case Reports* 2015; 3: 582–586.

79. Clara-Altamirano MA. Astragalus acrometastasis in endometrioid adenocarcinoma. *Gac Mex Oncol* 2016; 15: 43–46.

80. Devdas SK. Isolated humeral recurrence in endometrial carcinoma. *Indian J Med Paediatr Oncol* 2016; 37: 199–201.

81. Doǧr E. Bone metastasis in endometrial cancer: Evaluation of treatment approaches by factors affecting prognosis. *Eur J Gynaecol Oncol* 2016; 37: 407–416.

82. Liu A. Postoperative survival and functional outcomes for patients with metastatic gynecological cancer to the spine: case series and review of the literature. *J Neurosurg Spine* 2016; 24: 131–144.

83. Takeshita S. Thoracic laminectomy with spinal fixation in a nonambulatory patient with metastatic vertebral tumor from endometrial carcinoma. *J Obstet Gynaecol Res* 2016; 42: 1395–1399.

84. Casteillo F. TTF-1-positive Metastatic Endometrioid Carcinoma: A Case Report and Review of Literature of a Potential Diagnostic Pitfall. *Appl Immunohistochem Mol Morphol* 2017; 28: E6–E9.

85. Chin HX. Primary neuroendocrine carcinoma of the endometrium -a case report. *Eur J Gynaecol Oncol* 2017; 38: 818–820.

86. Soylemez MS. Endometrial adenocarcinoma recurrence presenting with tibial metastasis: Report of a case. *Int J Surg Case Rep* 2017; 36: 15–17.

87. Mahmood SS. Myocarditis with tremelimumab plus durvalumab combination therapy for endometrial cancer: A case report. *Gynecol Oncol Reports* 2018; 25: 74–77.

88. Sidibe FM. Small cell carcinoma of the endometrium: A clinicopathological study and management of three cases. *Bull Cancer* 2018; 105: 842–846.

89. Smith SH. A rare case of endometrial cancer metastatic to the uveal choroid. *Gynecol Oncol Reports* 2018; 23: 24–27.

90. Bashyam A. Metastatic endometrial cancer of the paranasal sinuses. *Ann R Coll Surg Engl* 2018; 100: e161–e164.

91. Makris G-M. Endometrial carcinoma with tibial bone metastasis: a case report and literature review. *J Obstet Gynaecol (Lahore)* 2018; 38: 1039–1047.

92. Adachi H. Precocious Solitary Cervical Metastasis from Endometrial Cancer Presenting as Cervical Radicular Pain. *World Neurosurg* 2018; 118: 162–167.

93. Dilek ON. Chest wall metastasis of endometrial cancer: case report and review of the literature. *Acta Chir Belg* 2019; 119: 243–247.

94. Madabhavi I V. Isolated acrometastasis: A rare presenting feature of endometrial carcinoma. *J Cancer Res Ther* 2019; 15: 1402–1404.

95. Su M. Adrenal and Bone Metastases as the Initial Presentation of Endometrial Carcinoma Diagnosed by 18F-FDG PET/CT. *Clin Nucl Med* 2020; 45: 711–713.

96. Najjar O. Aromatase inhibitor therapy in recurrent, estrogen-receptor positive uterine serous carcinoma: A case report. *Gynecol Oncol Reports*; 32. Epub ahead of print 2020. DOI: 10.1016/j.gore.2020.100555.

97. Aidos J. Endometrial carcinoma metastatic to the scalp. *Med*; 54. Epub ahead of print 2021. DOI: 10.11606/issn.2176-7262.rmrp.2021.166719.

98. Hayek J. Endometrial cancer recurrence in the sacrum and the Psoas: A case report and literature review. *Gynecol Oncol Reports*; 37. Epub ahead of print 2021. DOI: 10.1016/j.gore.2021.100814.

99. Tounsi N. Endometrial Carcinoma Presenting with an Isolated Osseous Metastasis: A Case Report. *Gynecol Obstet Reprod Med* 2021; 1–4.

100. Heidinger M. Endometrioid endometrial carcinoma of no-specific-molecular-profile with multiple bone metastases and muscle involvement: Case report and review of the literature. *Radiology case reports* 2022; 17: 2346–2352.

Table S1. Quality assessment according to the NIH Quality Assessment Tool for Observational Cohort and Cross-Sectional Studies

| **Study** | Question 1 | Question 2 | Question 3 | Question 4 | Question 5 | Question 6 | Question 7 | Question 8 | Question 9 | Question 10 | Question 11 | Question 12 | Question 13 | Question 14 |
| --- | --- | --- | --- | --- | --- | --- | --- | --- | --- | --- | --- | --- | --- | --- |
| **Kehoe**  **2010** | Yes | Yes | Yes | Yes | No | Yes | NA | NA | Yes | NA | Yes | No | Yes | Yes |
| **Blecharz**  **2011** | Yes | Yes | Yes | Yes | No | Yes | NA | NA | Yes | NA | Yes | No | Yes | No |
| **Uccella**  **2013** | Yes | Yes | Yes | Yes | No | Yes | NA | NA | Yes | NA | Yes | No | Yes | Yes |
| **Yoon**  **2014** | Yes | Yes | Yes | Yes | No | Yes | NA | NA | Yes | NA | Yes | No | Yes | Yes |
| **Kimyon**  **2016** | Yes | Yes | Yes | Yes | No | Yes | NA | NA | No | NA | Yes | No | Yes | No |
| **Liu**  **2019** | Yes | Yes | Yes | Yes | No | Yes | NA | NA | No | NA | Yes | No | Yes | Yes |
| **Ouldamer**  **2019** | Yes | Yes | Yes | Yes | No | Yes | NA | NA | Yes | NA | Yes | No | Yes | Yes |
| **Guo**  **2020** | Yes | Yes | Yes | Yes | No | Yes | NA | NA | No | NA | Yes | No | Yes | Yes |
| **McEachron**  **2020** | Yes | Yes | Yes | Yes | No | Yes | NA | NA | Yes | NA | Yes | No | Yes | Yes |
| **Hu**  **2021** | Yes | Yes | Yes | Yes | No | Yes | NA | NA | No | NA | Yes | No | Yes | Yes |
| **Yang**  **2021** | Yes | No | Yes | Yes | No | Yes | NA | NA | No | NA | Yes | No | Yes | Yes |
| **Wang**  **2022** | Yes | Yes | Yes | Yes | No | Yes | NA | NA | No | NA | Yes | No | Yes | No |

Question 1. Was the research question or objective in this paper clearly stated?

Question 2. Was the study population clearly specified and defined?

Question 3. Was the participation rate of eligible persons at least 50%?

Question 4. Were all the subjects selected or recruited from the same or similar populations (including the same time period)? Were inclusion and exclusion criteria for being in the study prespecified and applied uniformly to all participants?

Question 5. Was a sample size justification, power description, or variance and effect estimates provided?

Question 6. For the analyses in this paper, were the exposure(s) of interest measured prior to the outcome(s) being measured?

Question 7. Was the timeframe sufficient so that one could reasonably expect to see an association between exposure and outcome if it existed?

Question 8. For exposures that can vary in amount or level, did the study examine different levels of the exposure as related to the outcome (e.g., categories of exposure, or exposure measured as continuous variable)?

Question 9. Were the exposure measures (independent variables) clearly defined, valid, reliable, and implemented consistently across all study participants?

Question 10. Was the exposure(s) assessed more than once over time?

Question 11. Were the outcome measures (dependent variables) clearly defined, valid, reliable, and implemented consistently across all study participants?

Question 12. Were the outcome assessors blinded to the exposure status of participants?

Table S2. Quality assessment of case series and case reports according to the adapted Navigation Guide methodology according to Nambiema et al.

| **Autor** | Jahr | Question 1 | Question 2 | Question 3 | Question 4 | Question 5 | Question 6 | Question 7 | Question 8 |
| --- | --- | --- | --- | --- | --- | --- | --- | --- | --- |
| **Finn** | 1950 | No | Probably No | No | Probably No | No | No | No | No |
| **Milch** | 1953 | No | Probably No | No | Probably No | No | No | No | No |
| **Vanecko** | 1967 | No | Probably No | Probably No | Probably No | No | No | No | No |
| **Ravault** | 1967 | No | Probably No | Probably No | Probably Yes | No | No | No | No |
| **Gelbermann** | 1975 | No | Probably Yes | Probably Yes | Probably Yes | Probably Yes | No | No | No |
| **Boidi-trotti** | 1978 | No | Probably Yes | Probably Yes | Probably Yes | Probably No | No | No | No |
| **Beller** | 1982 | No | Probably Yes | Probably Yes | Probably Yes | Probably Yes | No | No | No |
| **Debois** | 1982 | No | Probably Yes | Probably Yes | Probably No | Probably No | No | No | No |
| **Onuba** | 1983 | No | Probably Yes | Probably Yes | Probably Yes | No | No | No | No |
| **Le loet** | 1983 | No | Probably No | Probably No | Probably No | Probably No | No | No | No |
| **Aalders** | 1984 | No | Probably Yes | Probably No | Probably No | No | No | No | No |
| **Kapp** | 1985 | No | No | Probably Yes | Probably No | Probably Yes | No | No | No |
| **Lieschke** | 1990 | No | No | Probably Yes | Probably No | Probably Yes | No | No | No |
| **Litton** | 1991 | No | No | Probably Yes | Probably No | Probably Yes | No | No | No |
| **Maxymiw** | 1991 | No | Probably Yes | No | Probably No | No | No | No | No |
| **Nishida** | 1994 | No | Probably Yes | Probably Yes | Probably Yes | Probably Yes | No | No | No |
| **Cooper** | 1994 | No | No | No | Probably No | No | No | No | No |
| **Schols** | 1995 | No | Probably No | No | Probably No | No | No | No | No |
| **Clarke** | 1996 | No | Probably Yes | Probably Yes | Probably Yes | Probably No | No | No | No |
| **Giardina** | 1996 | No | Probably Yes | Probably Yes | Probably Yes | Probably Yes | No | No | No |
| **Malicky** | 1997 | No | Probably No | Probably No | Probably No | Probably No | No | No | No |
| **Armentano** | 1997 | No | Probably Yes | Probably Yes | Probably Yes | Probably Yes | No | No | No |
| **Kushner** | 1997 | No | Probably Yes | No | Probably No | No | No | No | No |
| **Fukunaga** | 1997 | No | Yes | Probably No | Probably No | Probably No | No | No | No |
| **Dosoretz** | 1999 | No | Probably Yes | No | Probably Yes | No | No | No | No |
| **Rocha** | 2000 | No | Probably Yes | Yes | Probably Yes | Yes | No | No | No |
| **Benedicic** | 2002 | No | No | No | Probably No | No | No | No | No |
| **Sahinler** | 2001 | No | Probably Yes | No | Probably Yes | No | No | No | No |
| **Mustafa** | 2001 | No | Probably No | No | Probably No | No | No | No | No |
| **Manolitsas** | 2002 | No | Probably Yes | No | Probably No | No | No | No | No |
| **Neto** | 2002 | No | Probably Yes | No | Probably No | No | No | No | No |
| **Ali** | 2003 | No | Probably No | No | Probably No | No | No | No | No |
| **Dursun** | 2003 | No | Yes | No | Probably No | No | No | No | No |
| **Arnold** | 2003 | No | Probably Yes | Probably No | Probably No | Probably No | No | No | No |
| **Ilvan** | 2004 | No | No | No | No | No | No | No | No |
| **Amiot** | 2005 | No | Probably Yes | Yes | Probably No | Yes | No | No | No |
| **Frankel** | 2006 | No | Probably Yes | Yes | Probably No | Yes | No | No | No |
| **Dursun** | 2006 | No | Probably No | No | Probably No | No | No | No | No |
| **Loizzi** | 2006 | No | Probably Yes | Probably Yes | Probably No | Probably Yes | No | No | No |
| **Osanai** | 2006 | No | No | Probably No | No | No | No | No | No |
| **Uharcek** | 2006 | No | No | No | Probably No | No | No | No | No |
| **Haraguchi** | 2006 | No | Probably Yes | Probably No | Probably No | Probably No | No | No | No |
| **Giannakopoulos** | 2006 | No | Probably Yes | Probably No | Probably No | Probably No | No | No | No |
| **Al Salam** | 2006 | No | Probably Yes | Probably No | No | Probably No | No | No | No |
| **Kaya** | 2007 | No | Probably No | No | Probably No | No | No | No | No |
| **Ishibashi** | 2007 | No | Probably Yes | Probably Yes | No | No | No | No | No |
| **Qin** | 2008 | No | Probably Yes | No | Probably No | No | No | No | No |
| **Albareda** | 2008 | No | No | No | No | No | No | No | No |
| **Pakos** | 2009 | No | Probably No | Probably No | Probably No | Probably No | No | No | No |
| **Srikantia** | 2009 | No | Probably Yes | Probably Yes | Probably No | Probably Yes | No | No | No |
| **Oaknin** | 2010 | No | Probably Yes | No | Probably No | No | No | No | No |
| **Shigemitsu** | 2010 | No | No | No | Probably No | No | No | No | No |
| **Chan** | 2010 | No | Probably Yes | Probably No | Probably Yes | Probably No | No | No | No |
| **Artioli** | 2010 | No | Probably No | Yes | Probably No | Yes | No | No | No |
| **Gottwald** | 2011 | No | Probably No | No | Probably No | No | No | No | No |
| **Kurt** | 2011 | No | Yes | Probably Yes | Probably No | Probably Yes | No | No | No |
| **Jiang** | 2011 | No | No | No | No | No | No | No | No |
| **Brown** | 2011 | No | No | Probably Yes | No | Probably Yes | No | No | No |
| **Turner** | 2011 | No | Probably Yes | No | Probably No | No | No | No | No |
| **Pieters** | 2011 | No | Probably No | No | No | No | No | No | No |
| **Batista** | 2011 | No | Probably No | No | No | No | No | No | No |
| **Vizzielli** | 2012 | No | Probably No | No | No | No | No | No | No |
| **Gottwald** | 2012 | No | Probably Yes | Probably No | Probably No | Probably No | No | No | No |
| **Chen** | 2012 | No | No | No | Probably No | No | No | No | No |
| **Tang** | 2012 | No | No | No | No | No | No | No | No |
| **Chen** | 2013 | No | No | No | No | No | No | No | No |
| **Myriokefalitaki** | 2013 | No | No | No | No | No | No | No | No |
| **Nguyen** | 2013 | No | Probably Yes | No | Probably No | No | No | No | No |
| **Cecchi** | 2014 | No | No | No | No | No | No | No | No |
| **Gonzalez-Benitez** | 2014 | No | No | No | No | No | No | No | No |
| **Daga** | 2015 | No | Yes | Probably Yes | Probably No | Probably Yes | No | No | No |
| **Longo** | 2015 | No | Yes | Probably Yes | Probably No | Probably Yes | No | No | No |
| **Luedke** | 2015 | No | Yes | Yes | Probably No | No | No | No | No |
| **Lunardi** | 2015 | No | Yes | No | Probably No | No | No | No | No |
| **Boukhar** | 2015 | No | Probably Yes | Probably Yes | Probably No | Probably Yes | No | No | No |
| **Ghosh** | 2015 | No | Probably Yes | Probably No | Probably No | Probably No | No | No | No |
| **Clara-Altamirano** | 2015 | No | Probably No | No | Probably No | No | No | No | No |
| **Chow** | 2015 | No | Probably No | Probably No | No | No | No | No | No |
| **Devdas** | 2016 | No | Probably No | Probably Yes | No | Probably Yes | No | No | No |
| **Dogr** | 2016 | No | Probably Yes | No | No | No | No | No | No |
| **Takeshita** | 2016 | No | No | No | No | No | No | No | No |
| **Liu** | 2016 | No | Probably No | No | No | No | No | No | No |
| **Söylemez** | 2017 | No | Probably Yes | No | Probably No | No | No | No | No |
| **Casteillo** | 2017 | No | Probably Yes | No | Probably Yes | No | No | No | No |
| **Chin** | 2017 | No | Probably Yes | No | Probably No | No | No | No | No |
| **Bashyam** | 2018 | No | No | Probably No | No | Probably No | No | No | No |
| **Dilek** | 2018 | No | Probably Yes | Probably No | Probably No | Probably No | No | No | No |
| **Makris** | 2018 | No | Probably Yes | No | Probably No | No | No | No | No |
| **Mahmood** | 2018 | No | Probably Yes | No | Probably No | No | No | No | No |
| **Smith** | 2018 | No | No | No | No | No | No | No | No |
| **Sidibe** | 2018 | No | No | No | No | No | No | No | No |
| **Adachi** | 2018 | No | Probably Yes | No | Probably No | No | No | No | No |
| **Madabhavi** | 2019 | No | No | No | Probably No | No | No | No | No |
| **Najjar** | 2020 | No | Probably Yes | No | Probably No | No | No | No | No |
| **Su** | 2020 | No | Probably Yes | Probably No | Probably No | Probably No | No | No | No |
| **Aidos** | 2021 | No | Probably Yes | Probably No | No | Probably No | No | No | No |
| **Hayek** | 2021 | No | Probably No | No | No | No | No | No | No |
| **Heidinger** | 2022 | No | No | No | No | No | No | No | No |
| **Tounsi** | 2022 | No | Probably Yes | No | Probably No | No | No | No | No |

Question 1. Are the study groups at risk of not representing their source populations in a manner that might introduce selection bias?

Question 2. Were exposure/ Intervention (toxic, treatment) assessment methods lacking accuracy?

Question 3. Were outcome assessment methods lacking accuracy?

Question 4. Was potential confounding inadequately incorporated?

Question 5. Were incomplete outcome data inadequately addressed?

Question 6. Does the study report appear to have selective outcome reporting?

Question 7. Did the study receive any support from a company, study author, or other entity having a financial interest?

Question 8. Did the study appear to have other problems that could put it at a risk of bias?

Table S3. Patient characteristics of cases published as case reports and case series

| **Author** | **Year** | **Age at EC diagnosis** | **Histologic subtype**  **1) Adenocarcinoma**  **2) Non-Adenocarcinoma** | **Grade** | **FIGO stage at diagnosis** | **BM status**  **1) Singular 2) Multiple** | **Other Distant metastasis**  **0) No, 1) Yes** | **Lung metastasis**  **0) No, 1) Yes** | **Liver metastasis**  **0) No, 1) Yes** | **Brain metastasis**  **0) No, 1) Yes** | **Intraperitoneal extension**  **0) No, 1) Yes** | **Bone surgery for BM**  **0) No, 1) Yes** | **Chemotherapy after BM**  **0) No, 1) Yes** | **Hormonal therapy after BM**  **0) No, 1) Yes** | **Osteooncological therapy after BM**  **0) No, 1) Yes** | **LRT for BM**  **0) No, 1) Yes** | **Survival after EC diagnosis [months]** | **Survival after BM diagnosis [months]** | **Survival status**  **0) Alive 1) Dead** |
| --- | --- | --- | --- | --- | --- | --- | --- | --- | --- | --- | --- | --- | --- | --- | --- | --- | --- | --- | --- |
| **Finn^1^** | 1950 | n.A. | 1 | n.A. | n.A. | 2 | 0 | 0 | 0 | 1 | 0 | 1 | 0 | 0 | 0 | 1 | 80 | 78 | 0 |
| **Milch^2^** | 1953 | 57 | 1 | n.A. | 4 | 1 | 0 | 0 | 0 | 0 | 0 | 1 | 0 | 0 | 0 | 1 | 24 | 24 | 0 |
| **Vanecko^3^** | 1967 | 67 | 1 | n.A. | 1 | 1 | 0 | 0 | 0 | 0 | 0 | 0 | 0 | 0 | 0 | 1 | 29 | 12 | 0 |
| **Vanecko^3^** | 1967 | 54 | 2 | n.A. | 4 | 2 | 0 | 0 | 0 | 0 | 0 | 0 | 0 | 1 | 0 | 1 | 30 | 30 | 1 |
| **Ravault^4^** | 1967 | 61 | n.A. | n.A. | n.A. | 1 | 0 | 0 | 0 | 0 | 0 | 1 | 0 | 0 | 0 | 1 | 43 | 7 | 0 |
| **Gelbermann^5^** | 1975 | 64 | 1 | 1 | n.A. | 1 | 0 | 0 | 0 | 0 | 0 | 0 | 0 | 0 | 0 | 1 | 7 | 2 | 0 |
| **Boidi-trotti^6^** | 1978 | 55 | n.A. | n.A. | n.A. | 1 | 0 | 0 | 0 | 0 | 0 | 0 | 0 | 0 | 0 | 1 | 1 | 37 | 0 |
| **Beller^7^** | 1982 | 59 | 1 | 2 | 1 | 1 | 1 | 1 | 0 | 0 | 0 | 0 | 1 | 1 | 0 | 1 | 10 | 1 | 0 |
| **Debois^8^** | 1982 | 50 | 1 | 3 | 2 | 2 | 1 | 0 | 0 | 0 | 1 | 0 | 0 | 0 | 0 | 0 | 5 | 1 | 1 |
| **Debois^8^** | 1982 | 56 | 1 | 2 | 1 | 2 | 1 | 1 | 1 | 0 | 0 | 1 | 0 | 0 | 0 | 1 | 28 | 4 | 1 |
| **Le loet^9^** | 1983 | 62 | 1 | 3 | 4 | 1 | 0 | 0 | 0 | 0 | 0 | 0 | 0 | 1 | 0 | 1 | 7 | 7 | 0 |
| **Onuba^10^** | 1983 | 57 | 1 | n.A. | 4 | 1 | 1 | 1 | 0 | 0 | 0 | 0 | 0 | 0 | 0 | 0 | 13 | 13 | 1 |
| **Aalders^11^** | 1984 | 66 | n.A. | n.A. | 4 | 2 | 0 | 0 | 0 | 0 | 0 | 0 | 1 | 1 | 0 | 1 | 23 | 23 | 1 |
| **Aalders^11^** | 1984 | 70 | n.A. | n.A. | 4 | 1 | 1 | 1 | 0 | 0 | 1 | 0 | 0 | 1 | 0 | 1 | 28 | 28 | 1 |
| **Kapp^12^** | 1985 | 76 | 2 | 1 | 1 | 1 | 0 | 0 | 0 | 0 | 0 | 1 | 0 | 1 | 0 | 1 | 55 | 30 | 0 |
| **Lieschke^13^** | 1990 | 71 | 1 | 2 | 4 | 2 | 1 | 1 | 1 | 0 | 0 | 1 | 0 | 0 | 0 | 0 | 1 | 1 | 1 |
| **Maxymiw^14^** | 1991 | 63 | 1 | 3 | 3 | 2 | 1 | 0 | 0 | 0 | 0 | 0 | 0 | 0 | 0 | 1 | 7 | 3 | 1 |
| **Litton^15^** | 1991 | 55 | 1 | 2 | 1 | 1 | 0 | 0 | 0 | 0 | 0 | 1 | 0 | 0 | 0 | 1 | 34 | 10 | 0 |
| **Nishida^16^** | 1994 | 61 | 1 | 1 | 3 | 1 | 0 | 0 | 0 | 0 | 0 | 0 | 1 | 0 | 0 | 0 | 2 | 1 | 0 |
| **Cooper^17^** | 1994 | 59 | 2 | 2 | 4 | 1 | 0 | 0 | 0 | 0 | 0 | 0 | 1 | 1 | 0 | 1 | 60 | 60 | 0 |
| **Schols^18^** | 1995 | 66 | 1 | 3 | 1 | 1 | 0 | 0 | 0 | 0 | 0 | 1 | 0 | 1 | 0 | 1 | 42 | 24 | 0 |
| **Giardina^19^** | 1996 | 55 | 1 | 3 | 4 | 1 | 1 | 1 | 0 | 0 | 0 | 0 | 0 | 0 | 0 | 1 | 15 | 1 | 1 |
| **Clarke^20^** | 1996 | 55 | 1 | n.A. | n.A. | 1 | 1 | 1 | 0 | 0 | 0 | 1 | 0 | 0 | 0 | 1 | 54 | 36 | 1 |
| **Kushner^21^** | 1997 | 56 | 1 | 1 | 1 | 1 | 1 | 1 | 0 | 0 | 0 | 0 | 1 | 0 | 0 | 0 | 18 | 3 | 1 |
| **Malicky^22^** | 1997 | 44 | 1 | 2 | 4 | 1 | 0 | 0 | 0 | 0 | 0 | 0 | 1 | 1 | 0 | 1 | 24 | 24 | 0 |
| **Armentano^23^** | 1997 | 74 | 1 | n.A. | 1 | 1 | 0 | 0 | 0 | 0 | 0 | 0 | 0 | 0 | 0 | 0 | 145 | 145 | 1 |
| **Fukunaga^24^** | 1997 | 42 | 2 | 3 | 1 | 2 | 0 | 0 | 0 | 0 | 0 | 1 | 1 | 1 | 0 | 1 | 62 | 35 | 0 |
| **Dosoretz^25^** | 1999 | 71 | 1 | 2 | 4 | 2 | 0 | 0 | 0 | 0 | 1 | 1 | 1 | 0 | 0 | 1 | 15 | 15 | 0 |
| **Rocha^26^** | 2000 | 67 | 1 | n.A. | 4 | 2 | 1 | 1 | 0 | 0 | 0 | 1 | 1 | 0 | 0 | 0 | 69 | 9 | 1 |
| **Sahinler^27^** | 2001 | 67 | 1 | 3 | 1 | 2 | 0 | 0 | 0 | 0 | 0 | 0 | 0 | 0 | 0 | 1 | 6 | 2 | 1 |
| **Mustafa^28^** | 2001 | 45 | 1 | 2 | 1 | 1 | 1 | 1 | 0 | 0 | 1 | 1 | 0 | 1 | 0 | 0 | 42 | 6 | 1 |
| **Benedicic^29^** | 2002 | 61 | 1 | 1 | 4 | 1 | 0 | 0 | 0 | 0 | 0 | 1 | 1 | 1 | 0 | 0 | 96 | 96 | 0 |
| **Manolitsas^30^** | 2002 | 76 | 1 | 3 | 4 | 1 | 1 | 1 | 0 | 0 | 0 | 0 | 1 | 1 | 0 | 1 | 19 | 19 | 1 |
| **Neto^31^** | 2002 | 39 | 2 | 2 | 4 | 1 | 0 | 0 | 0 | 0 | 0 | 1 | 0 | 0 | 0 | 1 | 36 | 36 | 0 |
| **Dursun^32^** | 2003 | 51 | 1 | 3 | 3 | 2 | 1 | 0 | 0 | 0 | 0 | 0 | 0 | 0 | 0 | 1 | 7 | 6 | 0 |
| **Ali^33^** | 2003 | 77 | 1 | 3 | 1 | 1 | 1 | 1 | 0 | 0 | 0 | 1 | 0 | 1 | 0 | 1 | 40 | 16 | 0 |
| **Arnold^34^** | 2003 | 63 | 1 | 1 | 4 | 1 | 0 | 0 | 0 | 0 | 0 | 1 | 0 | 1 | 0 | 1 | 60 | 60 | 0 |
| **Ilvan^35^** | 2004 | 72 | 2 | 3 | 2 | 2 | 1 | 1 | 1 | 0 | 0 | 0 | 0 | 0 | 0 | 0 | 15 | 1 | 1 |
| **Kim^36^** | 2005 | 54 | 2 | 3 | 3 | 1 | 1 | 0 | 1 | 0 | 1 | 0 | 1 | 0 | 0 | 0 | 21 | 11 | 1 |
| **Amiot^37^** | 2005 | 86 | 1 | 3 | 3 | 2 | 1 | 1 | 0 | 0 | 0 | 1 | 0 | 0 | 0 | 0 | 19 | 9 | 0 |
| **Dursun^38^** | 2006 | 69 | 2 | 3 | 3 | 2 | 0 | 0 | 0 | 0 | 0 | 0 | 0 | 0 | 0 | 0 | 2 | 1 | 1 |
| **Loizzi^39^** | 2006 | 51 | 1 | 3 | 4 | 2 | 0 | 0 | 0 | 0 | 0 | 0 | 1 | 0 | 1 | 0 | 2 | 2 | 1 |
| **Loizzi^39^** | 2006 | 73 | 1 | 3 | 4 | 1 | 1 | 1 | 0 | 0 | 0 | 0 | 1 | 0 | 1 | 0 | 9 | 9 | 1 |
| **Osanai^40^** | 2006 | 68 | n.A. | 3 | 1 | 1 | 0 | 0 | 0 | 0 | 0 | 0 | 1 | 0 | 1 | 0 | 61 | 39 | 0 |
| **Giannakopoulos^41^** | 2006 | 68 | 1 | 3 | 4 | 1 | 0 | 0 | 0 | 0 | 0 | 0 | 1 | 0 | 0 | 0 | 48 | 48 | 1 |
| **Al-Salam^42^** | 2006 | 48 | 2 | 3 | 1 | 2 | 0 | 0 | 0 | 0 | 0 | 1 | 0 | 0 | 0 | 1 | 86 | 1 | 0 |
| **Uharcek^43^** | 2006 | 67 | 1 | 1 | 4 | 2 | 0 | 0 | 0 | 0 | 0 | 1 | 1 | 1 | 0 | 0 | 20 | 20 | 0 |
| **Haraguchi^44^** | 2006 | 87 | 1 | n.A. | n.A. | 1 | 0 | 0 | 0 | 0 | 0 | 1 | 0 | 0 | 0 | 0 | 168 | 60 | 1 |
| **Frankel^45^** | 2007 | 67 | n.A. | n.A. | n.A. | n.A. | 0 | 0 | 0 | 0 | 0 | 1 | n.A. | n.A. | n.A. | n.A. | n.A. | 9 | 0 |
| **Ishibashi^46^** | 2007 | 64 | 2 | 3 | 4 | 1 | 1 | 0 | 0 | 0 | 1 | 0 | 0 | 0 | 0 | 1 | 6 | 6 | 1 |
| **Kaya^47^** | 2007 | 70 | 1 | 1 | 4 | 1 | 0 | 0 | 0 | 0 | 0 | 0 | 0 | 1 | 0 | 0 | 47 | 47 | 0 |
| **Albareda^48^** | 2008 | 62 | 1 | 1 | 1 | 1 | 0 | 0 | 0 | 0 | 0 | 1 | 0 | 1 | 0 | 1 | 63 | 26 | 0 |
| **Qin^49^** | 2008 | 48 | 1 | 3 | 2 | 2 | 0 | 0 | 0 | 0 | 0 | 1 | 1 | 1 | 0 | 1 | 42 | 64 | 0 |
| **Srikantia^50^** | 2009 | 41 | 1 | 3 | 1 | 2 | 1 | 0 | 1 | 1 | 0 | 0 | 0 | 0 | 0 | 1 | 6 | 1 | 0 |
| **Pakos^51^** | 2009 | 62 | 1 | 3 | 2 | 1 | 1 | 0 | 0 | 0 | 0 | 1 | 0 | 0 | 0 | 0 | 31 | 24 | 0 |
| **Artioli^52^** | 2010 | 74 | 1 | 3 | 4 | 1 | 0 | 0 | 0 | 0 | 0 | 0 | 1 | 0 | 1 | 1 | 2 | 2 | 0 |
| **Oaknin^53^** | 2010 | 69 | 1 | 1 | 1 | 2 | 1 | 0 | 0 | 0 | 1 | 0 | 1 | 1 | 0 | 1 | 90 | 6 | 1 |
| **Shigemitsu^54^** | 2010 | 57 | 1 | 2 | 4 | 1 | 1 | 1 | 0 | 0 | 0 | 0 | 1 | 0 | 1 | 1 | 21 | 21 | 1 |
| **Chan^55^** | 2010 | 62 | 1 | n.A. | n.A. | 1 | 0 | 0 | 0 | 0 | 0 | 1 | n.A. | n.A. | n.A. | n.A. | 21 | 18 | 1 |
| **Turner^56^** | 2011 | 77 | 1 | n.A. | 4 | 2 | 0 | 0 | 0 | 0 | 0 | 0 | 0 | 1 | 0 | 1 | 1 | 1 | 1 |
| **Kurt^57^** | 2011 | 62 | 1 | 1 | 1 | 2 | 0 | 0 | 0 | 0 | 0 | 0 | 1 | 1 | 1 | 1 | 18 | 16 | 0 |
| **Gottwald^58^** | 2011 | 59 | 1 | 2 | 2 | 2 | 0 | 0 | 0 | 0 | 0 | 0 | 1 | 0 | 0 | 1 | 30 | 21 | 0 |
| **Pieters^59^** | 2011 | 76 | 1 | 2 | 2 | 2 | 1 | 0 | 1 | 0 | 0 | 0 | 1 | 1 | 0 | 1 | 36 | 33 | 1 |
| **Brown^60^** | 2011 | 62 | 1 | 2 | 1 | 2 | 1 | 0 | 1 | 0 | 0 | 1 | 1 | 0 | 0 | 0 | 36 | 1 | 0 |
| **Batista^61^** | 2011 | 65 | 2 | 3 | 1 | 1 | 0 | 0 | 0 | 0 | 0 | 1 | 0 | 0 | 0 | 1 | 168 | 24 | 0 |
| **Jiang^62^** | 2011 | 51 | 2 | 2 | 4 | 2 | 1 | 1 | 0 | 0 | 0 | 1 | 1 | 1 | 0 | 0 | 56 | 56 | 0 |
| **Gottwald^63^** | 2012 | 74 | 1 | 2 | 4 | 2 | 0 | 0 | 0 | 0 | 0 | 0 | 0 | 0 | 0 | 1 | 43 | 43 | 0 |
| **Tang^64^** | 2012 | 25 | 2 | 3 | 4 | 1 | 0 | 0 | 0 | 0 | 0 | 0 | 1 | 0 | 0 | 0 | 48 | 48 | 0 |
| **Chen^65^** | 2012 | 39 | 1 | 2 | 1 | 1 | 0 | 0 | 0 | 0 | 0 | 1 | 0 | 0 | 0 | 1 | 31 | 24 | 0 |
| **Vizzielli^66^** | 2012 | 62 | 1 | 2 | 4 | 1 | 1 | 1 | 0 | 0 | 0 | 1 | 1 | 0 | 0 | 0 | 30 | 30 | 0 |
| **Chen^67^** | 2013 | 61 | 2 | n.A. | 1 | 1 | 0 | 0 | 0 | 0 | 0 | 0 | 0 | 0 | 0 | 1 | 57 | 54 | 0 |
| **Nguyen^68^** | 2013 | 56 | 1 | 1 | 4 | 1 | 1 | 0 | 0 | 0 | 0 | 0 | 1 | 0 | 1 | 1 | 9 | 9 | 0 |
| **Myriokefalitaki^69^** | 2013 | 57 | 1 | 2 | 4 | 1 | 0 | 0 | 0 | 0 | 0 | 1 | 0 | 1 | 1 | 1 | 53 | 53 | 0 |
| **Gonzalez-Benitez^70^** | 2014 | 66 | 1 | 2 | 3 | 2 | 0 | 0 | 0 | 0 | 0 | 0 | 1 | 0 | 0 | 1 | 13 | 3 | 1 |
| **Cecchi^71^** | 2014 | 80 | 2 | 3 | 3 | 1 | 0 | 0 | 0 | 0 | 0 | 1 | 0 | 0 | 0 | 0 | 40 | 4 | 0 |
| **Luedke^72^** | 2015 | 64 | 2 | 3 | 4 | 2 | 0 | 0 | 0 | 0 | 0 | 0 | 1 | 0 | 0 | 1 | 1 | 1 | 0 |
| **Longo^73^** | 2015 | 62 | 1 | 2 | 4 | 1 | 1 | 1 | 0 | 0 | 0 | 0 | 1 | 0 | 1 | 1 | 4 | 4 | 0 |
| **Daga^74^** | 2015 | 75 | 1 | 2 | 4 | 2 | 0 | 0 | 0 | 0 | 0 | 0 | 0 | 1 | 1 | 1 | 6 | 6 | 0 |
| **Ghosh^75^** | 2015 | n.A. | 1 | 3 | n.A. | 1 | 0 | 0 | 0 | 0 | 0 | 0 | 1 | 0 | 0 | 1 | n.A. | 10 | 1 |
| **Ghosh^75^** | 2015 | n.A. | 1 | 3 | n.A. | 2 | 0 | 0 | 0 | 0 | 0 | 0 | 0 | 0 | 0 | 1 | n.A. | 11 | 1 |
| **Boukhar^76^** | 2015 | 77 | 2 | 2 | 4 | 2 | 1 | 1 | 0 | 0 | 0 | 0 | 0 | 1 | 0 | 0 | 24 | 24 | 0 |
| **Chow^77^** | 2015 | 53 | 2 | 3 | 1 | 2 | 1 | 1 | 0 | 0 | 0 | 0 | 1 | 0 | 0 | 1 | 264 | 44 | 1 |
| **Chow^77^** | 2015 | 38 | 2 | 3 | 1 | 1 | 0 | 0 | 0 | 0 | 0 | 1 | 1 | 0 | 0 | 1 | 132 | 24 | 0 |
| **Lunardi^78^** | 2015 | 63 | 1 | 3 | 3 | 2 | 0 | 0 | 0 | 0 | 0 | 1 | 1 | 0 | 0 | 1 | 24 | 24 | 0 |
| **Clara-Altamirano^79^** | 2016 | 57 | 1 | 2 | 3 | 1 | 0 | 0 | 0 | 0 | 0 | 0 | 1 | 0 | 1 | 1 | 12 | 6 | 0 |
| **Devdas^80^** | 2016 | 52 | 1 | 1 | 1 | 1 | 0 | 0 | 0 | 0 | 0 | 0 | 1 | 0 | 1 | 1 | 27 | 3 | 0 |
| **Dogr^81^** | 2016 | 80 | 1 | 2 | 4 | 1 | 1 | 1 | 0 | 0 | 0 | 1 | 1 | 1 | 0 | 0 | 10 | 10 | 1 |
| **Liu^82^** | 2016 | 48 | n.A. | n.A. | n.A. | 1 | 0 | 0 | 0 | 0 | 0 | 1 | 0 | 0 | 0 | 1 | 37 | 25 | 1 |
| **Liu^82^** | 2016 | 32 | n.A. | n.A. | n.A. | 1 | 0 | 0 | 0 | 0 | 0 | 1 | 0 | 0 | 0 | 1 | 76 | 28 | 0 |
| **Takeshita^83^** | 2016 | 64 | 1 | 2 | 4 | 1 | 1 | 1 | 0 | 0 | 0 | 1 | 1 | 0 | 0 | 1 | 45 | 45 | 0 |
| **Casteillo^84^** | 2017 | 74 | 1 | 3 | 1 | 2 | 1 | 1 | 0 | 0 | 0 | 0 | 1 | 0 | 0 | 0 | 15 | 3 | 0 |
| **Chin^85^** | 2017 | 54 | 2 | n.A. | 1 | 1 | 1 | 0 | 0 | 0 | 0 | 0 | 1 | 0 | 0 | 1 | 21 | 12 | 1 |
| **Söylemez^86^** | 2017 | 57 | 1 | n.A. | n.A. | 1 | 0 | 0 | 0 | 0 | 0 | 1 | 1 | 0 | 0 | 0 | 41 | 17 | 0 |
| **Mahmood^87^** | 2018 | 75 | 2 | n.A. | 3 | 2 | 1 | 1 | 1 | 0 | 0 | 0 | 1 | 0 | 0 | 0 | 9 | 3 | 0 |
| **Sidibe^88^** | 2018 | 47 | 2 | 3 | 4 | 2 | 1 | 1 | 1 | 0 | 0 | 0 | 1 | 0 | 0 | 0 | 8 | 8 | 1 |
| **Smith^89^** | 2018 | 75 | 1 | 3 | 1 | 2 | 1 | 1 | 0 | 1 | 0 | 0 | 1 | 0 | 0 | 0 | 60 | 39 | 1 |
| **Bashyam^90^** | 2018 | 59 | 1 | 2 | 3 | 1 | 1 | 0 | 1 | 0 | 1 | 1 | 0 | 0 | 0 | 1 | 49 | 1 | 0 |
| **Makris^91^** | 2018 | 68 | 1 | 1 | 4 | 1 | 0 | 0 | 0 | 0 | 0 | 1 | 1 | 0 | 0 | 0 | 6 | 6 | 0 |
| **Adachi^92^** | 2018 | 55 | 1 | 3 | 4 | 1 | 0 | 0 | 0 | 0 | 0 | 1 | 1 | 0 | 0 | 1 | 8 | 8 | 0 |
| **Dilek^93^** | 2019 | 57 | 1 | n.A. | 4 | 2 | 1 | 1 | 0 | 0 | 0 | 1 | 0 | 0 | 0 | 0 | 114 | 18 | 0 |
| **Madabhavi^94^** | 2019 | 66 | 1 | 1 | 4 | 1 | 0 | 0 | 0 | 0 | 0 | 0 | 1 | 0 | 1 | 1 | 6 | 6 | 0 |
| **Su^95^** | 2020 | 47 | 1 | 3 | 4 | 2 | 1 | 0 | 0 | 0 | 0 | 0 | 1 | 0 | 0 | 0 | 3 | 3 | 1 |
| **Najjar^96^** | 2020 | 61 | 2 | 3 | 3 | 2 | 1 | 1 | 0 | 0 | 0 | 0 | 1 | 1 | 1 | 0 | 138 | 30 | 0 |
| **Aidos^97^** | 2021 | 65 | 2 | 3 | 3 | n.A. | 1 | 1 | 0 | 1 | 0 | 0 | 0 | 0 | 0 | 0 | 23 | 2 | 1 |
| **Hayek^98^** | 2021 | 73 | 1 | 2 | 1 | 1 | 0 | 0 | 0 | 0 | 0 | 0 | 1 | 0 | 0 | 0 | 24 | 12 | 0 |
| **Tounsi^99^** | 2022 | 56 | 1 | 2 | 4 | 1 | 0 | 0 | 0 | 0 | 0 | 1 | 1 | 0 | 0 | 0 | 10 | 10 | 0 |
| **Heidinger^100^** | 2022 | 83 | 1 | 3 | 3 | 2 | 1 | 0 | 0 | 0 | 1 | 1 | 1 | 0 | 1 | 1 | 26 | 11 | 1 |

Table S4. Primary therapy for endometrial carcinoma in 134 patients with subsequent bone metastasis

| Treatment | n | % | Range in reported studies  ^24,26,29,41, Studies of table S3^ |
| --- | --- | --- | --- |
| Surgery | 19 | 14.2 | 0-25% |
| Surgery + Chemotherapy | 15 | 11.2 | 11-25% |
| Surgery + Chemotherapy + WPRT | 10 | 7.5 | 0-25% |
| Surgery + Chemotherapy + Vaginal Brachytherapy | 2 | 1.5 | 0-4% |
| Surgery + Chemotherapy + WPRT + Vaginal Brachytherapy | 5 | 3.7 | 0-25% |
| Surgery + WPRT | 30 | 22.4 | 0-48% |
| Surgery + Vaginal Brachytherapy | 10 | 7.5 | 0-33% |
| Surgery + WPRT + Vaginal Brachytherapy | 34 | 25.4 | 22-80% |
| Chemotherapy | 2 | 1.5 | 0-10% |
| Chemotherapy + WPRT | 1 | 0.7 | 0-5% |
| WPRT | 4 | 3.0 | 0-12% |

WPRT – Whole Pelvic radiotherapy
